# Adaptive evolution of electron transfer pathways in *Thermoanaerobacterium saccharolyticum*

**DOI:** 10.1128/jb.00057-26

**Published:** 2026-05-28

**Authors:** João H. T. M. Fabri, Shu Huang, T. Emme Burgin, Luana W. Bergamo, Lee R. Lynd, Daniel G. Olson

**Affiliations:** 1 Centro de Biologia Molecular e Engenharia Genética, Universidade Estadual de Campinas 28132, Campinas, Brazil; 2 Thayer School of Engineering at Dartmouth College, Hanover, New Hampshire, USA; 3 Terragia Biofuel Incorporated, Hanover, New Hampshire, USA; 4 Center for Bioenergy Innovation, Oak Ridge National Laboratory 6146, Oak Ridge, Tennessee, USA; University of Southern California, Los Angeles, California, USA

**Keywords:** electron transfer, spontaneous mutations, ferredoxin:NAD(P)H oxidoreductase, hydrogenase, alcohol dehydrogenase, pyruvate formate lyase

## Abstract

*Thermoanaerobacterium saccharolyticum* is an anaerobic, thermophilic bacterium that has been proposed for use in consolidated bioprocessing in coculture with cellulolytic bacteria such as *Clostridium thermocellum* for ethanol production. Although the mixed acid fermentation of both species has been engineered to produce ethanol as the major fermentation product, the maximum titer produced thus far by *C. thermocellum* is half that produced by *T. saccharolyticum*. This has motivated us to understand the mechanistic basis of the robust *T. saccharolyticum* ethanol pathway, so that key features can be recapitulated in *C. thermocellum*. Previously, we characterized the *individual* role of the main genes responsible for electron transfer in the ethanol production of *T. saccharolyticum*. However, the consequences of the *combined* loss of function of these genes has not been investigated, nor has the way in which fermentative metabolism adapts to such constraints. Here, we combined knockouts of ferredoxin nicotinamide oxidoreductases (*nfnA* and *nfnB*) and hydrogenases (*hydA* and *hfsD*). We showed that these genetic modifications together impair growth and decrease electron transfer from reduced ferredoxin, thereby redirecting flux from the pyruvate ferredoxin oxidoreductase enzyme to the pyruvate formate lyase enzyme. We also performed adaptive evolution of these mutants to rescue their growth and observed a mutation in the alcohol dehydrogenase *adhA* gene. We determined that this point mutation causes a structural change that impairs the AdhA specificity for the NADPH cofactor and increases NADH-linked activity to restore redox balance. These findings consolidate our understanding of electron transfer pathways in this organism.

**IMPORTANCE:**

*Thermoanaerobacterium saccharolyticum* has the potential to be used for the conversion of lignocellulose-derived sugars into bioethanol via consolidated bioprocessing in cocultures with *Clostridium thermocellum* or via transfer of its ethanol production pathway to the same bacterium. However, attempts to transfer the pathway to increase ethanol titer have not yet been successful. A deeper understanding of how electron transfer pathways operate in this thermophile and how they adapt to metabolic disturbances (such as the absence of metabolic pathways for ferredoxin oxidation, for example) will improve our ability to engineer this organism for increased product formation and improve our ability to transfer its remarkable ethanol production phenotype to other microbes. These, in turn, have potential benefits for sustainable production of fuels and chemicals.

## INTRODUCTION

Cellulosic biofuels (referred to as second-generation biofuels to be distinguished from first-generation biofuels produced from starch and sugar) are likely essential for achieving deep decarbonization of the global transport sector (1). The conventional method of producing second-generation ethanol is based on the use of soluble sugar-fermenting organisms (e.g., *Saccharomyces cerevisiae* or *Zymomonas mobilis*), thermochemical pretreatment, and the addition of fungal enzymes and is still too expensive for widespread commercial application (2–6). Consolidated bioprocessing (CBP) has been proposed as an alternative approach that has the potential to lower the cost of lignocellulose processing, due, among other reasons, to the use of engineered thermophilic bacteria that natively ferment lignocellulose (7).

Although *Clostridium thermocellum* is a promising candidate for CBP, it does not ferment five-carbon (C5) sugars present in hemicellulose, such as xylose and arabinose (8, 9). Furthermore, the best engineered strains so far can produce ethanol at titers of 25–30 g/L (10, 11), which is lower than the 40–50 g/L titers thought to be necessary for stand-alone facilities (6, 12). One alternative is *Thermoanaerobacterium saccharolyticum*, which has already been engineered for ethanol production at high titers (70 g/L) (13). This anaerobic thermophilic bacterium is capable of fermenting C5 and C6 sugars and soluble C5 oligomers (i.e., xylan) from lignocellulosic biomass, although it cannot break down cellulose (14–16). To enable cellulosic ethanol production, we have proposed transferring the ethanol production pathway from this organism to native cellulolytic organisms such as *C. thermocellum*. However, previous efforts resulted in only moderate increases in the ethanol titer (17, 18), suggesting that our current understanding of the ethanol production pathway in *T. saccharolyticum* is incomplete.

**Fig 1:**
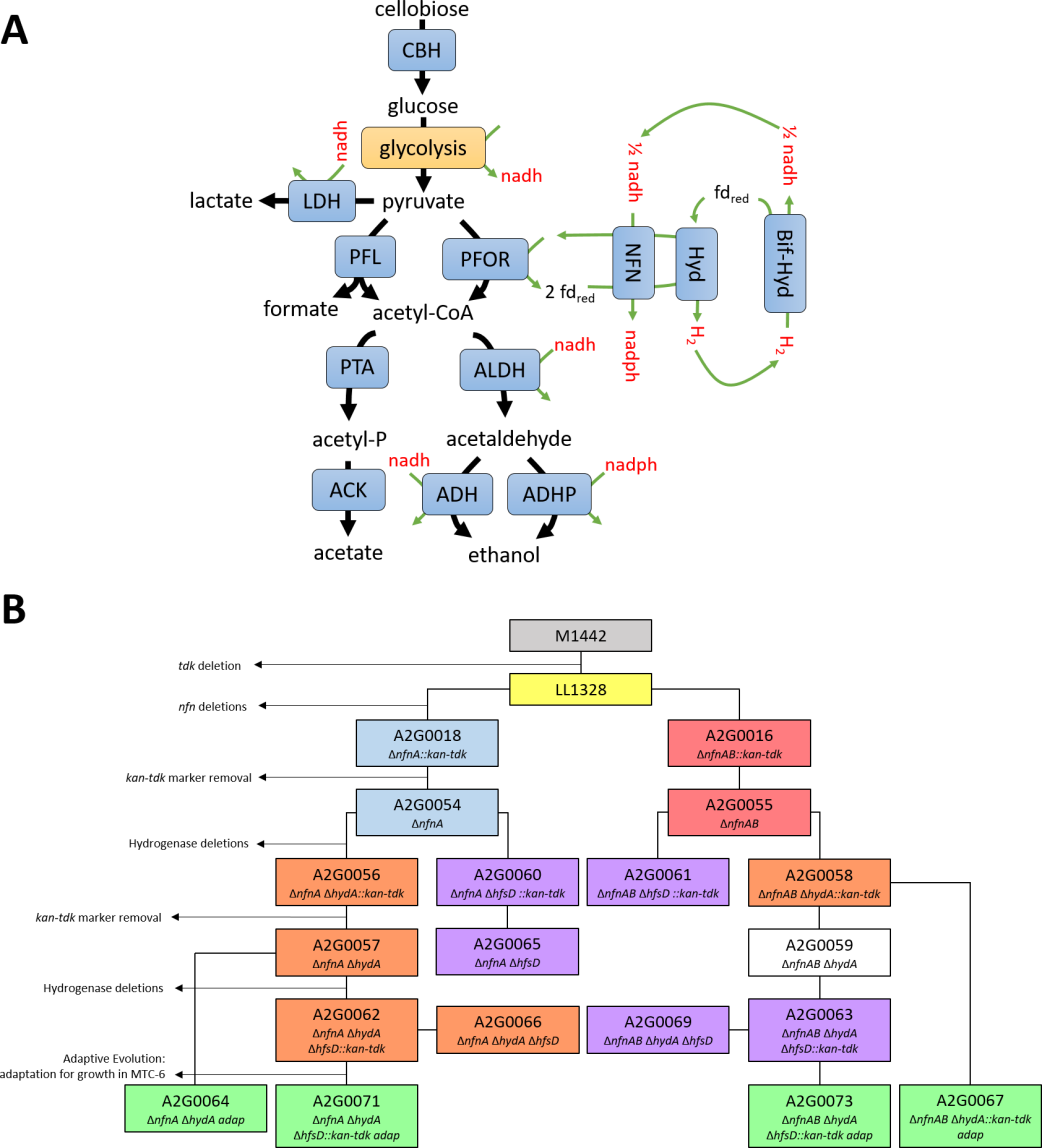
*T. saccharolyticum* wild-type metabolic pathways and strain construction diagram. (**A**) Black arrows represent carbon conversions, and green arrows represent electron transfer. For redox cofactor pairs (NADH/NAD^+^, NADPH/NADP^+^, and Fd_red_/Fd_ox_), only the reduced form is shown to avoid clutter. Enzyme abbreviations include cellobiose hydrolase (CBH), lactate dehydrogenase (LDH), pyruvate formate lyase (PFL), pyruvate ferredoxin oxidoreductase (PFOR), phosphotransacetylase (PTA), acetate kinase (ACK), acetaldehyde dehydrogenase (ALDH), NADH-linked alcohol dehydrogenase (ADH), NADPH-linked alcohol dehydrogenase (ADHP), NADH-dependent reduced ferredoxin:NADP^+^ reductase (NFN), electron-bifurcating hydrogenase (Bif-Hyd), and ferredoxin hydrogenase (Hyd). The Hyd reaction, mediated by the *hfsD* gene, proceeds in the forward direction to generate hydrogen from reduced ferredoxin. This hydrogen is then taken up by the reversible bifurcating hydrogenase (Bif-Hyd), mediated by the *hydABCD* gene cluster. Detailed stoichiometry of each reaction is shown in Supporting Table S6. (**B**) Strategy for construction of the strains: the original homoethanologenic strain, M1442, is shown in gray. A descendant with a *tdk* deletion (used for counter-selection with FUDR) is shown in yellow. The lineage with *nfnA* only deletions is shown in light blue. The lineage with *nfnAB* deletions is shown in red. Strains that cannot grow on the defined MTC-6 medium are shown in orange. Strains obtained after adaptive evolution to improve growth on MTC-6 are shown in green. Strains with the highest PFL flux, excluding the adapted ones, are shown in purple. Full genotypes of the strains are listed in Table 1.

The *T. saccharolyticum* ethanol pathway allows efficient transfer of electrons from carbohydrate to ethanol (19–21). In this organism, pyruvate from glycolysis is converted to acetyl-CoA, either via the pyruvate ferredoxin oxidoreductase (PFOR) or pyruvate formate lyase (PFL) reactions (Fig. 1A). Next, acetyl-CoA is converted to acetaldehyde via the acetaldehyde dehydrogenase (ALDH) reaction (via AdhE) and finally to ethanol via the alcohol dehydrogenase (ADH) reaction (via AdhE and/or AdhA) (19, 22). While flux through the PFL reaction generates formate as a byproduct (supplying C1 units for biosynthesis) (23), flux through PFOR reaction generates reduced ferredoxin (Fd_red_), which must be reoxidized to allow the PFOR reaction to proceed. Usually, this reoxidation is performed with ferredoxin:NAD(P)H oxidoreductases (Fnors), i.e., NfnA and NfnB, which catalyze electron transfer from ferredoxin to the nicotinamide cofactor NADPH (19, 24). Interestingly, multiple hydrogenases working in opposite directions can allow chemical transformations equivalent to that of an FNOR reaction in a process called hydrogen cycling (19, 20). In this proposed system, the HfsD hydrogenase operates in the forward direction to generate hydrogen from reduced ferredoxin. This hydrogen is then taken up by the reversible bifurcating hydrogenase (Bif-Hyd, HydABCD) to produce NADH and regenerate reduced ferredoxin, which could be consumed by NfnAB to produce NADPH (Fig. 1A and Supporting Table S6). Recently, we characterized knockout mutants and identified the genes *nfnA*, *nfnB*, *hydA*, and *hfsD* as essential for high ethanol yield in *T. saccharolyticum* (19). However, the overlapping roles of these enzymes in electron transfer left open the possibility of additional, unknown electron transfer mechanisms. In this work, we performed cumulative deletions of electron transfer pathways to demonstrate the absence of cryptic electron transfer pathways for Fd_red_ (or put upper bounds on the magnitude of fluxes through these pathways) and understand how the cell adapts to the absence of these pathways.

In this work, we combined Fnor and hydrogenase deletions and investigated the effects of these modifications on *T. saccharolyticum* growth and fermentation. One goal of this work was to create a strain of *T. saccharolyticum* where FNOR activity is linked to growth. To compensate for the deleterious effects of the knockouts, we performed adaptive evolution of the most growth-compromised strains and identified and characterized the most common spontaneous mutations that arose during this process. Our results corroborated previous findings and confirmed the importance of Fnors and hydrogenases for the growth and fermentative metabolism of *T. saccharolyticum*, as well as showing how its ethanol production pathway adapts in the absence of these enzymes.

## MATERIALS AND METHODS

### Strain construction

The strains used in this study are listed in Table 1, and the primers used for construction and confirmation of the mutant strains are listed in Supporting Table S1. All the genetic modifications were introduced into the *T. saccharolyticum* chromosome by homologous recombination and using the kanamycin (*kan*) resistant gene as a marker. The thymidine kinase (*tdk*) gene was used as the negative selection marker, allowing a second round of transformation when using 5-fluoro-2′-deoxyuridine (FUDR) to remove the *kan-tdk* marker, as described previously (25, 26). The knockout cassettes consisted of the *kan-tdk* marker flanked by upstream and downstream homology regions of the target genes (*nfnA*, *nfnAB*, *hydA*, *hfsD, gntR,* or *phoU*). The insertion cassette containing an *adhA* point mutation consisted of four independent fragments: the non-mutated 5′ region of the *adhA* gene, a variable region of the *adhA* gene containing the G50D mutation, the *kan-tdk* marker, and the downstream homology region of the *adhA* gene. Both knockout and insertion cassettes were constructed from linear PCR products via Gibson assembly (27). *T. saccharolyticum* transformation was conducted by incubating the cassettes along with the cells at 55°C to allow the naturally competent *T. saccharolyticum* cells to take up the DNA constructs (28). The transformants were selected in agar plates supplemented with 200 µg/mL kanamycin or, in the case of marker removal transformations, different concentrations of FUDR (10–50 µg/mL).

**TABLE 1:** *T. saccharolyticum* strains used in this study

Strain	Parent strain	Genotype	Source	Sequencing data
LL1025	None, WT	Wild-type *T. saccharolyticum* JW/SL-YS485	(29)	GenBank accession no. CP003184.
M1442	LL1025	LL1025 Δ*pta* Δ*ack* Δl*dh adhE*^G544D^	Also called LL1049 (13)	SRA accession no. SRA233073.
LL1328	M1442	M1442 Δ*tdk*	(19)	SRA accession no. SRR37575190.
A2G0016	LL1328	M1442 Δ*tdk* Δ*nfnAB::kan-tdk*	(19)	SRA accession no. SRR32258720.
A2G0018	LL1328	M1442 Δ*tdk* Δ*nfnA::kan-tdk*	(19)	SRA accession no. SRR32258718.
A2G0020	LL1328	M1442 Δ*tdk* Δ*hfsD::kan-tdk*	(19)	SRA accession no. SRR32258716.
A2G0054	A2G0018	M1442 Δ*tdk* Δ*nfnA*	This work	SRA accession no. SRR35227153.
A2G0055	A2G0016	M1442 Δ*tdk* Δ*nfnAB*	This work	SRA accession no. SRR35227152.
A2G0056	A2G0054	M1442 Δ*tdk* Δ*nfnA* Δ*hydA::kan-tdk*	This work	SRA accession no. SRR35227141.
A2G0057	A2G0056	M1442 Δ*tdk* Δ*nfnA* Δ*hydA*	This work	SRA accession no. SRR35227139.
A2G0058	A2G0055	M1442 Δ*tdk* Δ*nfnAB* Δ*hydA::kan-tdk*	This work	SRA accession no. SRR35227138.
A2G0059	A2G0058	M1442 Δ*tdk* Δ*nfnAB* Δ*hydA*	This work	SRA accession no. SRR35227137.
A2G0060	A2G0054	M1442 Δ*tdk* Δ*nfnA* Δ*hfsD::kan-tdk*	This work	SRA accession no. SRR35227136.
A2G0061	A2G0055	M1442 Δ*tdk* Δ*nfnAB* Δ*hfsD::kan-tdk*	This work	SRA accession no. SRR35227135.
A2G0062	A2G0057	M1442 Δ*tdk* Δ*nfnA* Δ*hydA* Δ*hfsD::kan-tdk*	This work	SRA accession no. SRR35227134.
A2G0063	A2G0059	M1442 Δ*tdk* Δ*nfnAB* Δ*hydA* Δ*hfsD::kan-tdk*	This work	SRA accession no. SRR35227133.
A2G0065	A2G0060	M1442 Δ*tdk* Δ*nfnA* Δ*hfsD*	This work	SRA accession no. SRR36260912.
A2G0066	A2G0062	M1442 Δ*tdk* Δ*nfnA* Δ*hydA* Δ*hfsD*	This work	SRA accession no. SRR36260911.
A2G0069	A2G0063	M1442 Δ*tdk* Δ*nfnAB* Δ*hydA* Δ*hfsD*	This work	SRA accession no. SRR36260910.
A2G0064-1	A2G0057	M1442 Δ*tdk* Δ*nfnA* Δ*hydA adap* 1	This work	SRA accession no. SRR35227151.
A2G0064-2	A2G0057	M1442 Δ*tdk* Δ*nfnA* Δ*hydA adap* 2	This work	SRA accession no. SRR35227150.
A2G0067-1	A2G0058	M1442 Δ*tdk* Δ*nfnAB* Δ*hydA::kan-tdk adap 1*	This work	SRA accession no. SRR35227149.
A2G0067-2	A2G0058	M1442 Δ*tdk* Δ*nfnAB* Δ*hydA::kan-tdk adap 2*	This work	SRA accession no. SRR35227148.
A2G0067-3	A2G0058	M1442 Δ*tdk* Δ*nfnAB* Δ*hydA::kan-tdk adap 3*	This work	SRA accession no. SRR35227147.
A2G0067-4	A2G0058	M1442 Δ*tdk* Δ*nfnAB* Δ*hydA::kan-tdk adap 4*	This work	SRA accession no. SRR35227146.
A2G0071-1	A2G0062	M1442 Δ*tdk* Δ*nfnA* Δ*hydA* Δ*hfsD::kan-tdk adap 1*	This work	SRA accession no. SRR35227145.
A2G0071-2	A2G0062	M1442 Δ*tdk* Δ*nfnA* Δ*hydA* Δ*hfsD::kan-tdk adap 2*	This work	SRA accession no. SRR35227144.
A2G0073-1	A2G0063	M1442 Δ*tdk* Δ*nfnAB* Δ*hydA* Δ*hfsD::kan-tdk adap 1*	This work	SRA accession no. SRR35227143.
A2G0073-2	A2G0063	M1442 Δ*tdk* Δ*nfnAB* Δ*hydA* Δ*hfsD::kan-tdk adap 2*	This work	SRA accession no. SRR35227142.
A2G0073-3	A2G0063	M1442 Δ*tdk* Δ*nfnAB* Δ*hydA* Δ*hfsD::kan-tdk adap 3*	This work	SRA accession no. SRR35227140.
A2G0070	A2G0020	M1442 Δ*tdk* Δ*hfsD*	This work	SRA accession no. SRR36260909.
A2G0074	LL1328	M1442 Δ*tdk* Δ*gntR::kan-tdk*	This work	SRA accession no. SRR36260908.
A2G0075	LL1328	M1442 Δ*tdk* Δ*phoU::kan-tdk*	This work	SRA accession no. SRR36260907.
A2G0076	LL1328	M1442 Δ*tdk adhA*::*adhA*^G50D^*-kan-tdk*	This work	SRA accession no. SRR36260906.
A2G0072	LL1328	M1442 Δ*tdk adhA*::*adhA*^WT^*-kan-tdk*	This work	SRA accession no. SRR36260905.

To express AdhA in *E. coli*, the genomic sequence of the *T. saccharolyticum adhA* gene (Tsac_2087) was PCR-amplified from strain LL1328 and cloned via Gibson assembly into plasmid pCB17 in place of the *C. thermocellum adhE* gene, generating plasmid pJHF01 (Table 2). *adhA* containing the G50D mutation was amplified from strain A2G0064 and was also cloned into plasmid pCB17 in place of the *C. thermocellum adhE* gene, generating plasmid pJHF02. Both vectors were transformed into *E. coli* ElectroMAX DH10ꞵ by electroporation (Invitrogen), following the manufacturers’ protocol.

**TABLE 2:** Plasmids used in this study

Name	Description	Source	GenBank accession number
pCB17/pLL1423	*C. thermocellum* AdhE N-histag	(30)	PX858269
pJHF01	*T. saccharolyticum* wild-type *adhA,* N-histag	This work	PX657364
pJHF02	*T. saccharolyticum* G50D mutated *adhA,* N-histag	This work	PX657365

### Media and growth conditions

All *T. saccharolyticum* strains were grown anaerobically at 55°C inside a COY anaerobic chamber (Coy Laboratory Products, Grass Lake, MI) using two different media. M122C-rich medium at pH 6.5 was used for *T. saccharolyticum* transformation and growth analysis (31). The chemically defined MTC-6 medium at pH 6.5 was used for end product and growth analyses (23) and was prepared with 20 g/L cellobiose as the main carbon source. For fermentation, a 1% inoculum from frozen cell culture was added to tubes containing 20 mL of MTC-6 medium, and these were incubated without shaking for 7 days at 55°C. For gas chromatography analysis, fermentation was performed in 125 mL glass serum bottles containing 25 mL of MTC-6 medium and incubated in an orbital shaking incubator for 7 days at 55°C and 250 rpm. To analyze the growth rates, cells were grown in 200 μL of MTC-6 or M122C in a 96-well plate (1% inoculum), and the absorbance at 600 nm was monitored every 8 min for 96 h using a BioTek Epoch2 microplate reader (Agilent). Growth in the presence of the pyruvate-formate lyase (PFL) inhibitor sodium hypophosphite (HPP) was assessed by adding different concentrations of HPP in a 96-well plate containing M122C or MTC-6 medium (32), and readings were taken as described above. For the growth tests, maximum optical density (OD_600_) values and growth rates were used to analyze and compare the strains. Maximum OD values were extracted from the growth curves by simply correcting for the baseline and taking the highest OD value achieved during the entire growth period. Growth rate values were calculated using two arbitrary OD values at time points within the exponential growth phase of each strain. To avoid the need to include different antibiotic controls for various strains, we did not use kanamycin or FUDR in the fermentation, growth, and HPP testing experiments. *E. coli* cells used for protein expression were grown in lysogeny broth (LB) medium.

### Fermentation end product analysis

After fermentation, 1 mL aliquots of each culture were centrifuged, the supernatants were acidified with 50 µL of 10% H_2_SO_4_, and then filtered using 0.2 µm PES syringe filters. Fermentation substrate and product concentrations were quantified using a Shimadzu LC-2050C high-performance liquid chromatograph (HPLC) coupled to an HPX-87H column and an additional UV detector. The column was incubated at 60°C, and the mobile phase (H_2_SO_4_ 2.5 mM) flow rate was set to 0.6 mL/min. All fermentation data are reported in Supporting Table S2. Material balance calculations were performed by combining data from HPLC, GC, and NMR measurements, as described previously (19).

### Gas chromatography (GC) analysis

The concentration of molecular hydrogen inside the bottles after fermentation was analyzed using an SRI 310C gas chromatograph (SRI Inc.), utilizing a HayeSep D packed column and nitrogen as carrier gas at a flow rate of 8.2 mL/min, as previously described (22). The column was incubated at 151°C, and the thermal conductivity detector (TCD) current was set to 80 mA. The absolute H_2_ measurement was determined by comparing peak heights to a known standard (ultrapure H_2_ 99.9999%, ALG Gases, Brazil) and after correction for pressure differences between the bottle (elevated pressure) vs the GC (atmospheric pressure). All GC data are reported in Supporting Table S3.

### Nuclear magnetic resonance (NMR) analysis

Measurement of additional fermentation products that were not detected by HPLC was performed by nuclear magnetic resonance (NMR) analysis. Samples were prepared as described in reference 19. The nuclear magnetic resonance experiment was carried out at LNBio (CNPEM, Campinas, Brazil), using a Varian/Agilent DD2 spectrometer (Agilent Technologies Inc., Santa Clara, CA, USA). Data acquisition parameters, data processing, and compound determination and quantification were performed as described in reference 19. All NMR data are reported in Supporting Table S4.

### Adaptive evolution

The adaptation process consisted of at least 10 serial transfers in MTC-6 defined medium containing decreasing concentrations of M122C-rich medium (starting with 5% of M122C and 95% of MTC-6), until the M122C medium was completely eliminated. Transfers were approximately 1% by volume (100 μL into 10 mL), which allows for approximately 6.6 generations per transfer, as previously described (30).

### Whole-genome sequencing

The strains were grown in M122C-rich medium, and cells were resuspended in 500 µL of Zymo 1× DNA/RNA Shield Ready-to-use (Zymo Research) in a 2 mL screw cap tube. DNA extraction and genome sequencing were performed by Plasmidsaurus using a hybrid sequencing approach with Oxford Nanopore sequencer and Illumina NextSeq2000 system (Plasmidsaurus Inc., California, USA), with custom analysis and annotation. Short reads were submitted to the NCBI’s Short Read Archive under the Bioproject PRJNA1314240. *De novo* genome assembly was done using *T. saccharolyticum* JW/SL-YS485 genomic sequence (NCBI reference sequence NC_017992.1) as reference in Geneious Prime 2024.0.5 software.

### Cloning, expression, purification, and enzymatic activity of AdhA

The plasmids harboring the wild-type and mutated AdhA proteins were cloned in chemocompetent *E. coli* T7 Express *lysY/I^q^* (New England Biolabs), following the manufacturers’ protocol. For protein expression, cells were grown under agitation at 37°C to an OD_600_ of 0.6 before protein expression induction with 0.4 mM of IPTG at 30°C overnight under anaerobic conditions. The cells were then pelleted and stored at −80°C. Cell pellets were resuspended in equilibration buffer (20 mM Tris, 500 mM NaCl, 10 mM imidazole, 1 mM dithiothreitol [DTT], pH 8.0) and lysed by sonication. Recombinant AdhA proteins were purified on a HisTrap HP column (Cytiva) using an ÄKTA start chromatography system. After clarification, the lysate was loaded onto the pre-equilibrated column. The column was washed with 10 column volumes (CV) of wash buffer (20 mM Tris, 500 mM NaCl, 30 mM imidazole, 1 mM DTT, pH 8.0) to remove nonspecifically bound proteins. Elution was performed with a linear gradient over 20 CV, increasing the imidazole concentration from 30 mM to 300 mM. Fractions corresponding to the protein peak were pooled and concentrated using Amicon Ultra centrifugal filters (MWCO 30 kDa). All steps were carried out anaerobically unless otherwise indicated. The ADH reaction was assayed in the physiological (ethanol-forming) direction and was carried out anaerobically at 55°C. The reaction mixture consisted of 0.3 mM NADH or NADPH, 30 mM acetaldehyde, 100 mM Tris-HCl (pH 7.5), 5 µM FeSO_4_, 1 mM DTT, and cell extract. ADH activity was monitored by measuring the absorbance at 340 nm using an Agilent 8453 UV-Visible Spectroscopy System. Protein concentration was determined by the Bradford method using bovine serum albumin (BSA) as the standard. ADH activity was calculated as previously described (31). One unit of activity (U) is equal to the formation of 1 µmol of product per minute. Specific activities are expressed in U per milligram of purified protein.

### Molecular dynamics simulations

Molecular dynamics simulations of wild-type and mutated variants of AdhA and GntR were conducted using Amber22 (33). Models were prepared based on AlphaFold Protein Structure Database entries AF-I3V X 46-F1-v6 and AF-I3VTC6-F1-v6 for AdhA and GntR, respectively (34, 35). Residue protonation states were predicted at pH 6.5 using H++ (36). Each protein was modeled using the ff19SB force field (37) and solvated in an octagonal box of OPC3 waters (38) with at least 8 Å between the edges of the protein and the nearest edge of the box, with sodium counterions added as needed to neutralize the overall charge. The NADPH cofactor in AdhA was initially positioned by STAMP structural alignment (39, 40) with the AdhE-NAD complex in PDB ID: 7BVP and was modeled according to the parameters from reference (41). Hydrogen mass repartitioning with a factor of 3 was then applied to enable a longer simulation timestep (42). All simulations were conducted using an Andersen thermostat (43) with a time constant of 1,000 steps, an 8 Å non-bonded cutoff radius, and a time step of 4 fs unless otherwise described. Energy minimization was performed over 5,000 steps, followed by heating at constant volume from 100 to 310.15 K over 11,000 2-fs steps. Then, models were equilibrated at constant pressure for 10 ns. After equilibration, mutations were applied as needed using PyRosetta (44), followed by protonation state predictions from PROPKA3 (45). Independent copies of production simulations were performed for each variant, including the wild type: eight 100 ns simulations for AdhA and four 400-ns simulations for GntR. Visualizations were made with VMD (46).

## RESULTS

### The combination of *fnor* and *hydrogenase* knockouts impairs *T. saccharolyticum* growth, which was recovered through adaptive evolution

Maintenance of redox homeostasis is necessary for growth. In *T. saccharolyticum*, catabolism of sugars via glycolysis generates NADH, which must be oxidized to maintain redox homeostasis. In addition, flux through the PFOR reaction generates Fd_red_, which also must be oxidized to maintain redox homeostasis (Fig. 1A). In the *T. saccharolyticum* M1442 strain, carbon and electron flux has been directed toward ethanol production, resulting in a homoethanologenic phenotype (13). Previously, we observed that individually deleting the *fnor* and *hydrogenase* genes in our homoethanologenic strain impacts both the ethanol titer and growth, demonstrating that the four genes (*nfnA*, *nfnB*, *hfsD*, and *hydA*) are all necessary for high-yield ethanol production (19). Aiming to better understand how electron transfer works in *T. saccharolyticum* and particularly how these genes interact with each other, as well as whether any additional cryptic electron transfer genes are present, we combined the knockouts of four genes (*nfnB*, *nfnA*, *hydA*, and *hfsD*) in different ways (Fig. 1B).

**Fig 2:**
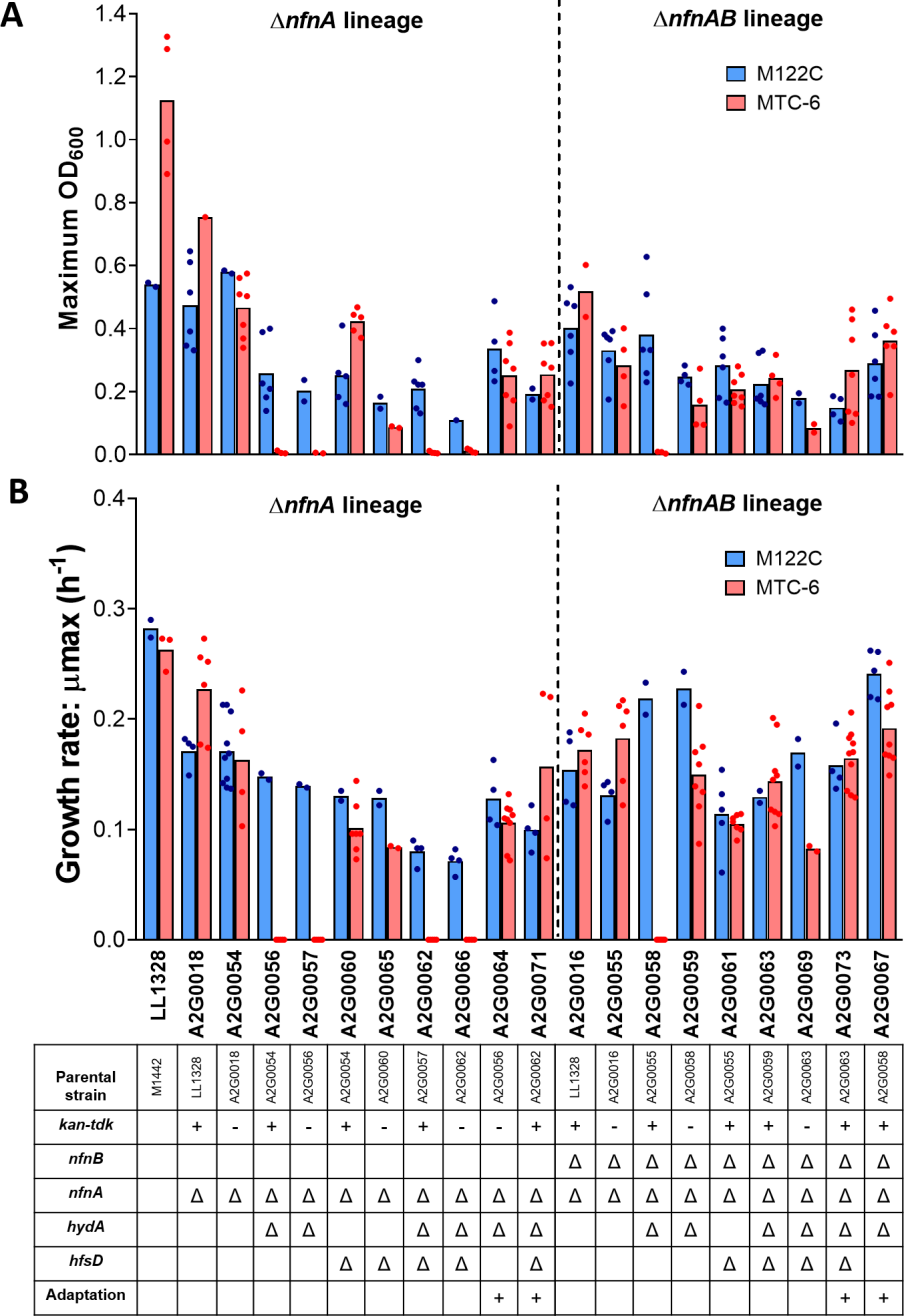
Growth phenotype of the strains. (**A**) Maximum OD and (**B**) growth rate of each strain in defined (MTC-6) and rich (M122C) media. The strains were cultivated in a 96-well plates containing MTC-6 medium or M122C medium for 96 h at 55°C. Readings were taken every 8 min. Dots indicate biological replicates. The dashed line separates the strains according to the parental lineage: on the left are the strains derived from Δ*nfnA* lineage, and on the right are the strains derived from Δ*nfnAB* lineage. The genotype of each strain is shown in the table below the graphics, where: blanks indicate the WT alleles; Δ indicates disruption of the gene by replacement with a *kan-tdk* marker; for the *kan-tdk* marker line, + indicates the presence and - indicates the absence of the marker; and for the adaptation line, + indicates the presence of adaptation and blanks indicate the absence of adaptation.

To combine the knockouts, starting from strain LL1328, which is derived from the M1442 ethanologenic strain by the deletion of the *tdk* gene, two lineages were created: one derived from the *nfnA* knockout and the other from the *nfnAB* knockout. This was done so that we could better study the role of *nfnB*, considered the main Fnor enzyme of *T. saccharolyticum*. Next, we removed the *kan-tdk* marker through markerless transformation using FUDR and deleted the hydrogenases *hydA*, *hfsD*, or both, repeating the marker removal step after each knockout. The resulting strains were named as shown in Fig. 1B and Table 1 and were subjected to a growth analysis over a 96-hour period in both M122C-rich and MTC-6 defined media (Supporting Fig. S1). The deletion of *nfnA* and *nfnAB*, as previously seen, is sufficient to decrease the growth of *T. saccharolyticum*, reducing the maximum optical density (OD) and the growth rate achieved in both media, with more severe inhibition for the *nfnAB* deletion (Fig. 2). In defined medium, for example, the maximum OD of the Δ*nfnA* and Δ*nfnAB* strains were 0.8 and 0.5, respectively, while the LL1328 parental strain reached an OD of 1.15. The subsequent deletion of hydrogenases is even more detrimental, retarding the growth of several strains and further reducing the maximum OD and growth rate achieved by many of them (max OD ≤ 0.4 and growth rate ≤ 0.17, both in MTC-6, for all the strains). Despite this, all mutants were still able to grow in the rich M122C medium, but five were unable to grow in defined MTC-6 medium: the Δ*nfnA* Δ*hydA* strain (both with [A2G0056] or without [A2G0057] the *kan-tdk* marker), Δ*nfnAB* Δ*hydA* (A2G0058, with the *kan-tdk* marker), and Δ*nfnA* Δ*hydA* Δ*hfsD* strain (both with [A2G0062] or without [A2G0066] the *kan-tdk* marker). Note that we do not expect the presence of the *kan-tdk* marker to affect the growth phenotype. Thus, the fact that the Δ*nfnAB* Δ*hydA kan-tdk* strain was unable to grow in MTC-6 but regained that ability after removal of the *kan-tdk* marker suggests that this may be due to some other mutation acquired during the process of marker removal.

Since some strains grew only in rich medium (M122C), but not in chemically defined medium (MTC-6), we performed serial transfers with a gradually decreasing proportion of rich medium until the cells were growing in 100% chemically defined medium. Thus, strain A2G0057 (Δ*nfnA* Δ*hydA*) generated strain A2G0064 (Δ*nfnA* Δ*hydA* adap) after adaptation; strain A2G0062 (Δ*nfnA* Δ*hydA* Δ*hfsD::kan-tdk*) generated strain A2G0071 (Δ*nfnA* Δ*hydA* Δ*hfsD::kan-tdk* adap); strain A2G0058 (Δ*nfnAB* Δ*hydA::kan-tdk*) generated strain A2G0067 (Δ*nfnAB* Δ*hydA::kan-tdk* adap); and strain A2G0063 (Δ*nfnAB* Δ*hydA* Δ*hfsD::kan-tdk*) generated strain A2G0073 (Δ*nfnAB* Δ*hydA* Δ*hfsD::kan-tdk* adap), as shown in Fig. 1B. This last strain, despite being able to grow in MTC-6, was chosen for adaptation and comparison with the others, considering that it is the strain harboring the full set of four cumulative deletions. As can be seen in Fig. 2, evolutionary adaptation allowed all adapted strains to grow in MTC-6, but growth in M122C-rich medium was largely unaffected.

### Effect of combining knockouts and adaptive evolution on fermentation

**Fig 3:**
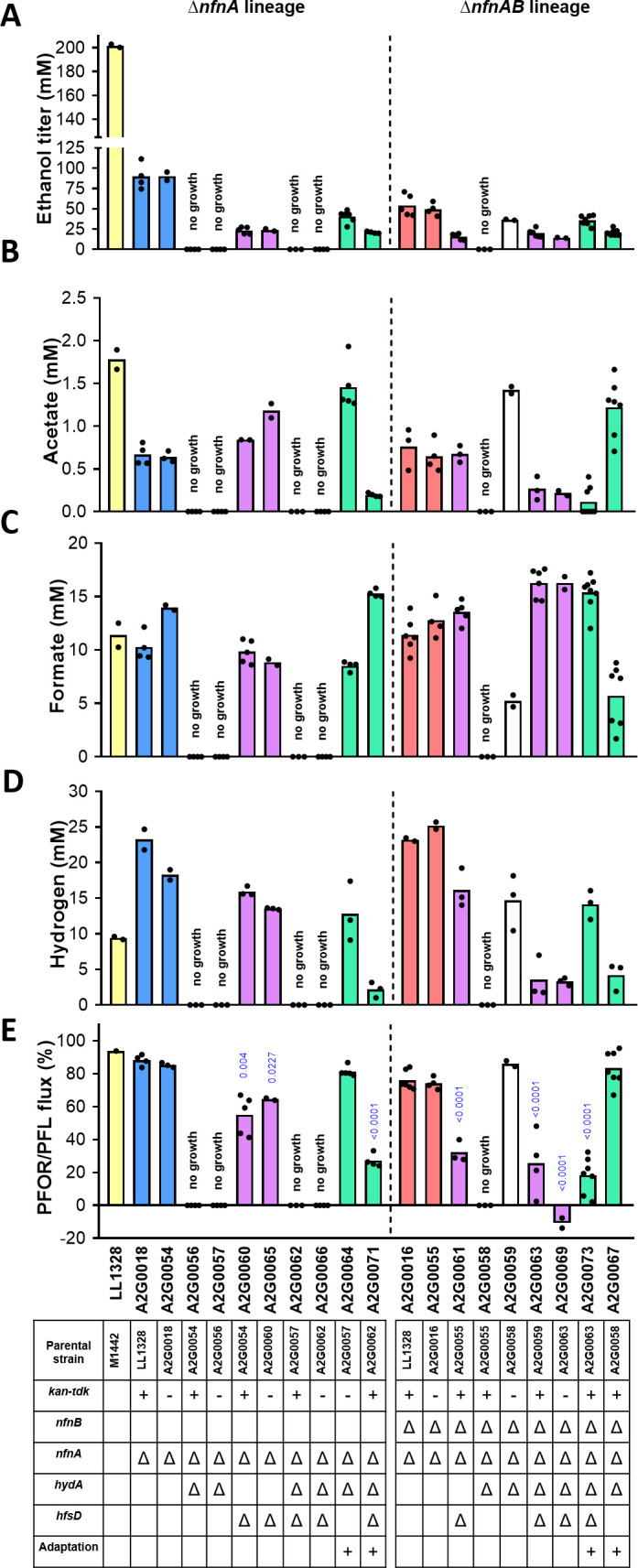
Fermentation products of the mutant strains. (**A–C**) Ethanol, acetate, and formate titers. (**D**) Hydrogen production. Hydrogen gas is reported in units of mmol headspace H_2_ per L of fermentation volume (mM) for ease of comparison with other measurements. (**E**) PFOR/PFL flux, calculated by the ratio (acetate and ethanol)/formate. In this panel, one-way ANOVA and Dunnett’s post-tests were performed to determine the significance of decreased PFOR/PFL flux ratio compared to the starting strain (LL1328) (blue text above each bar). All the strains were cultivated in MTC-6 defined medium containing 20 g/L of cellobiose for 7 days. Black dots indicate the biological replicates. The colors of the bars match the colors from Fig. 1. The dashed line separates the strains according to the parental lineage: on the left are the strains derived from Δ*nfnA* lineage, and on the right are the strains derived from Δ*nfnAB* lineage. The genotype of each strain is shown in the table below the graphics, where: blanks indicate the WT alleles; Δ indicates disruption of the gene by replacement with a *kan-tdk* marker; for the *kan-tdk* marker line, + indicates the presence and - indicates the absence of the marker; and for the adaptation line, + indicates the presence of adaptation and blanks indicate the absence of adaptation. All fermentation data are reported in Supporting Table S2. All GC data are reported in Supporting Table S3.

Previously, we found that the single deletion of *nfnA*, *nfnAB*, *hydA*, or *hfsD* considerably reduces the ethanol yield and titer of *T. saccharolyticum* fermentations (19). To better understand the physiological significance of cumulative deletions, both before and after adaptive evolution, all the mutant strains were submitted to HPLC analysis after fermentation and compared to the original parental strain (the LL1328 homoethanologenic strain). As expected, all the mutant strains exhibited low ethanol titer (Fig. 3A), and in general, additional deletions further reduced ethanol titer. The Δ*nfnA* strain (both with and without the *kan* marker, A2G0018 and A2G0054, respectively) showed a 50% reduction in ethanol titer compared to the LL1328 strain. Subsequent deletion of *hfsD* (Δ*nfnA* Δ*hfsD* strains with and without the *kan* marker, A2G0060 and A2G0065) resulted in a total reduction of 88% in ethanol titer. Since the fermentation was carried out in defined MTC-6 medium, ideal for quantifying fermentation products, strains unable to grow in this medium could not be analyzed. However, both adapted strains from the Δ*nfnA* lineage—the double deletion Δ*nfnA* Δ*hydA* (A2G0064) and triple deletion Δ*nfnA* Δ*hydA* Δ*hfsD::kan-tdk* (A2G0071)—by regaining growth on MTC-6, began to produce ethanol, but at very low levels (80% and 90% reduction, respectively, compared to the LL1328 ethanologenic strain). Regarding the Δ*nfnAB* strain (both with and without the *kan* marker, A2G0016 and A2G0055, respectively), the reduction in ethanol titer was approximately 75%, which was expected. Subsequent deletion of *hfsD* (Δ*nfnAB* Δ*hfsD::kan-tdk* strain, A2G0061) resulted in a total reduction of 92% in ethanol titer. The Δ*nfnAB* Δ*hydA* strain without the *kan* marker (A2G0059) showed an 82% reduction in ethanol titer, while the subsequent deletion of *hfsD* (Δ*nfnAB* Δ*hydA* Δ*hfsD* strains with and without the *kan* marker, A2G0063 and A2G0069) resulted in a reduction of more than 90% in ethanol titer, which was not altered after adaptation (A2G0073 strain). However, the adaptation of the Δ*nfnAB* Δ*hydA* strain (A2G0058) resulted in a strain (A2G0067) capable of growing in defined media and producing ethanol, with a titer comparable to that of the Δ*nfnAB* Δ*hydA* strain without the *kan* marker (A2G0059). Acetate, another product of *T. saccharolyticum* fermentation, was produced at relatively low levels (<2 mM) by all strains, including the parent strain (Fig. 3B). This is expected because all of these strains are descended from strain M1442, which has a deletion of the *pta* and *ack* genes (Fig. 1A), the primary acetate production pathway.

In the ethanol production pathway of *T. saccharolyticum*, two enzymes are capable of converting pyruvate into acetyl-CoA: Pfor and Pfl (23). Flux through the Pfl enzyme can be directly observed via formate production (Fig. 3C). By contrast, flux through the Pfor reaction has to be estimated based on the other fluxes (acetate, ethanol, formate). The Fd_red_ generated by the Pfor reaction has to be oxidized via conversion to nicotinamide cofactors (i.e., FNOR activity) or production of hydrogen (i.e., hydrogenase activity). Since hydrogen can be measured directly (Fig. 3D), it allows for a rough estimate of Fnor flux. Given that all of the genes we are studying in this work (*nfnA*, *nfnB*, *hfsD*, and *hydA*) interact with ferredoxin, we hypothesized that cumulative deletion of all of them would block flux through the PFOR reaction. Then, we calculated the ratio between the relative fluxes of PFOR and PFL (Fig. 3E) following the equation:


PFOR/PFL flux ratio = [1−formate/(acetate+ ethanol)]∗100%.

Flux ratio values close to 0% indicate dominance of the PFL reaction (and low prevalence of the PFOR reaction), while values close to 100% indicate dominance of the PFOR reaction (and low prevalence of the PFL reaction). Although deletion of individual electron transfer genes (*nfnA*, *nfnB*, *hfsD*, or *hydA*) dramatically decreased ethanol flux, the PFOR/PFL ratio was more resistant to change, requiring at least two mutations (i.e., *nfnA* or *nfnAB* plus *hfsD*) to see a significant change. The Δ*nfnAB* lineage was more negatively affected than the Δ*nfnA* lineage. Furthermore, the deletion of *hfsD* was associated with a stronger reduction in PFOR flux compared to *hydA*. The Δ*nfnA* Δ*hydA* strains (A2G0056 and A2G0057) were initially unable to grow in defined medium, but after adaptation (strain A2G0064), showed PFOR/PFL flux ratios similar to the starting strain (LL1328). Similarly, the Δ*nfnAB* Δ*hydA::kan-tdk* strain was initially unable to grow in defined medium, but after marker removal (strain A2G0059) or serial transfer (strain A2G0067, Δ*nfnAB* Δ*hydA::kan-tdk* adap), growth was restored, and PFOR/PFL flux ratios were similar to the starting strain (LL1328) (47).

In strains where *hfsD* had already been deleted, the further deletion of *hydA* further decreased the PFOR/PFL ratio, as expected. Note that because the PFOR flux is calculated based on formate, acetate, and ethanol measurements, as the ratio approaches zero (i.e., formate production is approximately equal to acetate plus ethanol production), small measurement errors can cause relatively large fluctuations in the calculated value, including negative values, as is seen for strain A2G0069 (Δ*nfnAB* Δ*hydA* Δ*hfsD*). In these cases, it can be useful to consider hydrogen production as well. All of the hydrogenases in *T. saccharolyticum* are thought to use Fd_red_ for at least some of the electrons transferred to hydrogen (48). For strains with deletions of all four target genes (*nfnA*, *nfnB*, *hfsD*, and *hydA*), the low levels of hydrogen production combined with low levels of PFOR/PFL ratio (that fluctuate around zero) suggest that flux through the Pfor enzyme has been almost completely eliminated.

Considering only the hydrogen production gives us another way to understand electron transfer in *T. saccharolyticum*. The impact of the combined knockouts on molecular hydrogen production was also investigated through GC analysis (Fig. 3D). All the *nfn* deletions resulted in increased hydrogen levels (from 2.5 to 3.0 times higher than the LL1328 parental strain), very similar to previous results (19). Subsequent deletion of *hydA* or *hfsD* reduced these values, while triple and quadruple mutants (either before or after adaptive evolution) produced even less hydrogen (50 to 80% less), which must be a direct effect of the loss of function of the hydrogenases.

**Fig 4:**
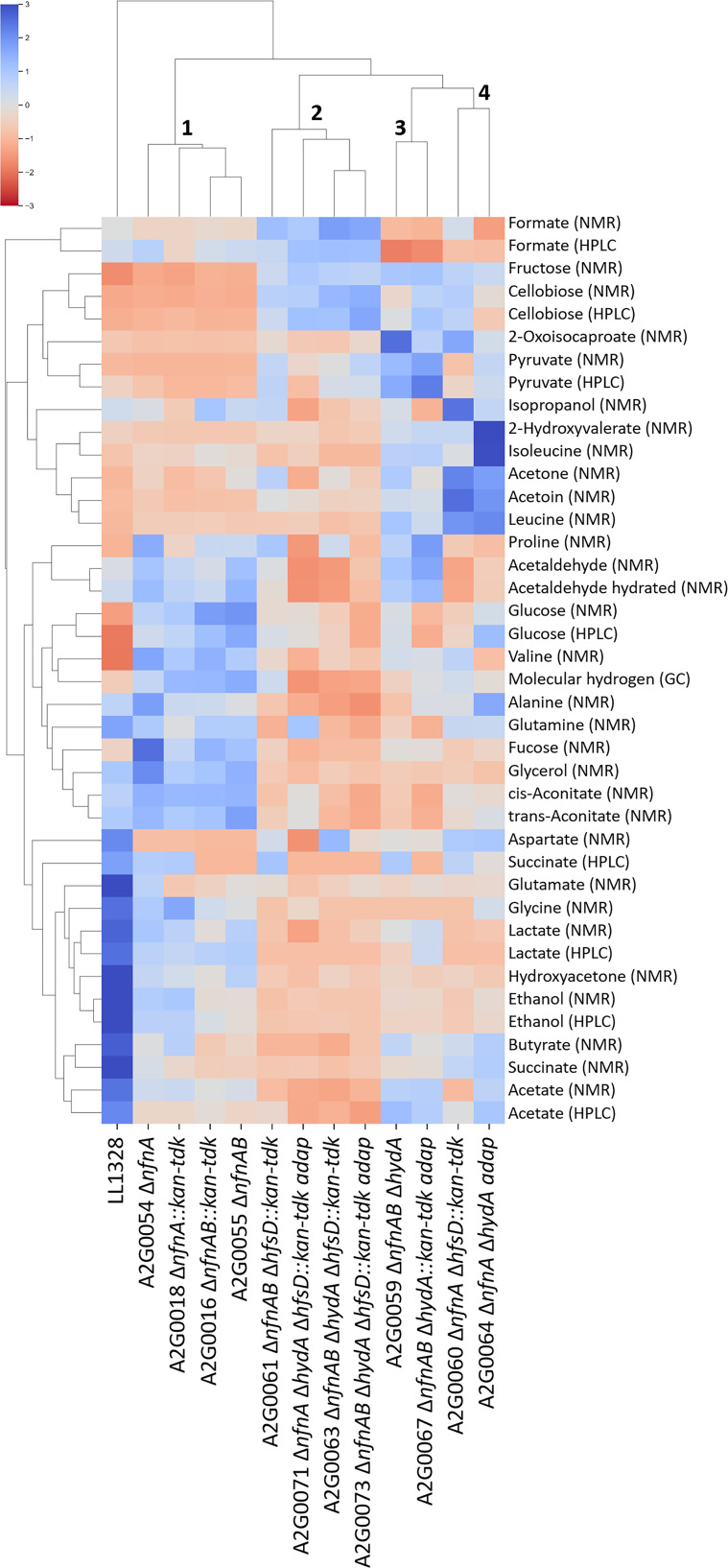
HPLC, NMR, and GC analyses of fermentation products of the mutant strains. All the strains were cultivated in MTC-6 defined medium containing 20 g/L of cellobiose for 7 days. Samples were filtered and subjected to HPLC, NMR, or GC analysis. NMR: duplicates; HPLC and GC: two or more replicates. The values underwent Z-score normalization and were hierarchically clustered using Ward’s linkage method with the seaborn clustermap (Python). The numbers above the heat map indicate the four clusters discussed in the text. All NMR data are reported in Supporting Table S4.

To gain a broader understanding of the effect of the knockouts on the metabolism of *T. saccharolyticum*, we submitted the fermentation samples of the mutant strains to nuclear magnetic resonance (NMR) analysis in order to identify and quantify metabolites not measured by HPLC. The strains (only the ones that grow in defined medium) and quantified metabolites were clustered and arranged in a heat map shown in Fig. 4, where HPLC and GC data were also included for a more complete analysis. All mutant strains presented lower levels of the fermentation products ethanol, acetate, lactate, butyrate, and succinate, as well as the amino acids aspartate, glutamate, and glycine, in comparison to the LL1328 ethanologenic ancestor strain. In addition, the mutant strains clustered into four specific groups. The first cluster includes the single mutants Δ*nfnA* and Δ*nfnAB*, both with and without the *kan* marker (strains A2G0016, A2G0018, A2G0054, and A2G0055). These strains accumulated glucose (derived from the cellobiose used as substrate), some amino acids such as proline and valine, and small amounts of acetaldehyde and aconitate.

The second cluster contains the Δ*nfnA* Δ*hydA* Δ*hfsD::kan-tdk* adapted (A2G0071), Δ*nfnAB* Δ*hfsD* (A2G0061), and Δ*nfnAB* Δ*hydA* Δ*hfsD::kan-tdk* strains, both before and after adaptation (A2G0063 and A2G0073, respectively). These strains clearly failed to consume a significant portion of the cellobiose from the medium and also exhibited lower levels for almost all metabolites compared to the other strains. However, they accumulated much more formate, consistent with the results shown in Fig. 3, suggesting again a deviation of the pyruvate to acetyl-CoA flux from PFOR to PFL reaction.

The third cluster contains the Δ*nfnAB* Δ*hydA* (A2G0059) and Δ*nfnAB* Δ*hydA::kan-tdk* adapted (A2G0067) strains, whose main characteristic was to accumulate more acetaldehyde and pyruvate, in addition to acetoin. Production of acetaldehyde can be used to reoxidize the NADH generated by glycolysis, which may not be able to be converted to ethanol due to a lack of NADPH.

Finally, the fourth cluster contains the Δ*nfnA* Δ*hfsD::kan-tdk* (A2G0060) and Δ*nfnA* Δ*hydA* adapted (A2G0064) strains. These mutants also consumed less cellobiose than those in cluster 1 and did not produce as much glucose. At the same time, they did not accumulate as much acetaldehyde as clusters 1 and 3, but they showed higher levels of the amino acids leucine and isoleucine, and several intermediates or byproducts from the biosynthesis pathways for these amino acids, including 2-oxoisocaproate, 2-hydroxyvalerate, and acetoin. Many of the genes for these pathways are found in a putative operon spanning genes Tsac_0563 to Tsac_0570, including two enzymes that may affect redox balance: 3-isopropylmalate dehydrogenase (EC 1.1.1.85, Tsac_0568), which produces NADH in the physiological direction, and ketol-acid reductoisomerase (EC 1.1.1.86, Tsac_0564), which consumes NADPH in the physiological direction. Changes in expression of these genes may provide the organisms with alternative means of maintaining redox balance.

**Fig 5:**
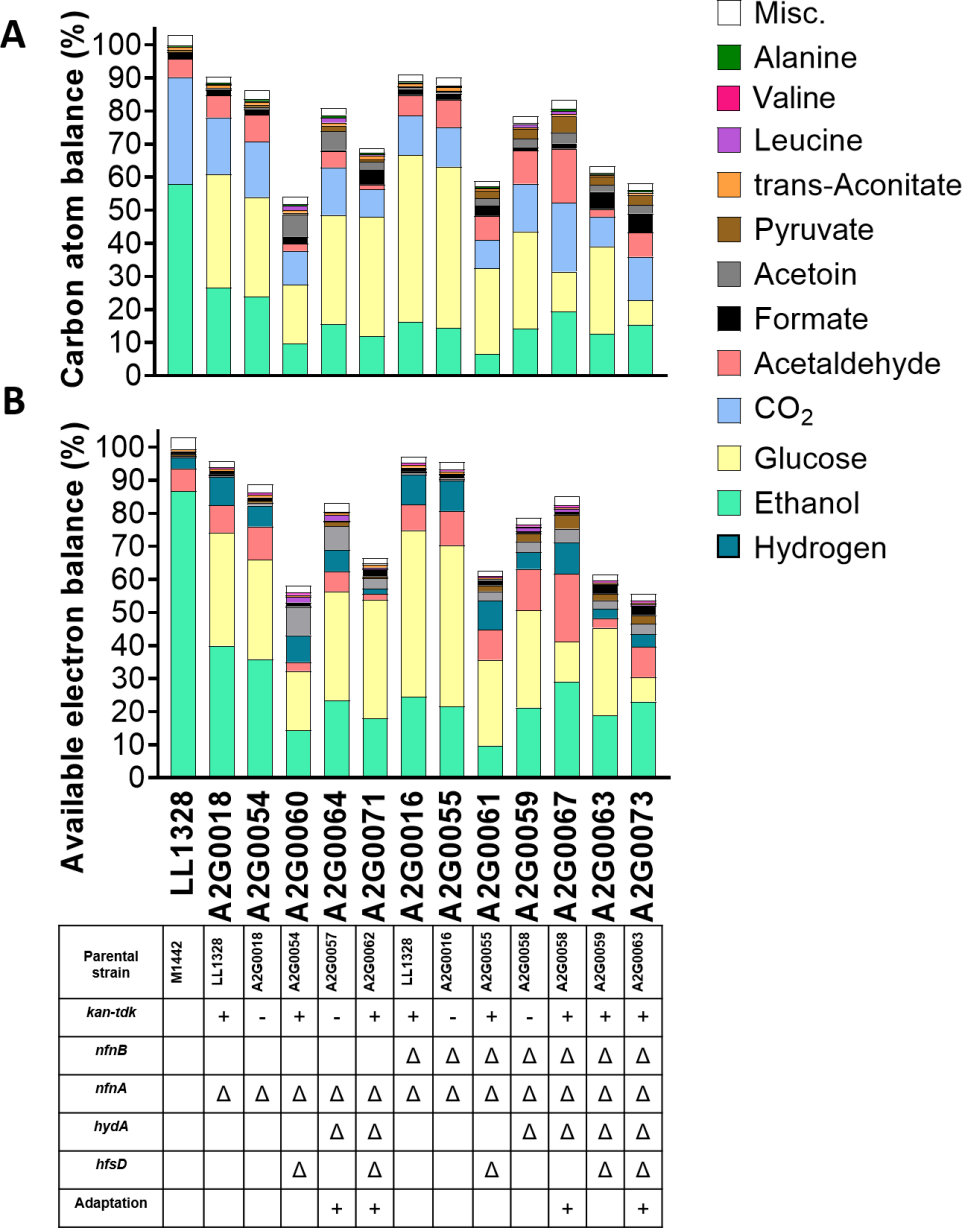
Carbon and available electron balances of the mutant strains. The calculation was performed using values obtained from HPLC, GC, and NMR analyses. For both carbon (**A**) and electron (**B**) balances, data for the top 10 compounds are presented individually, and the remaining compounds are lumped together as “Misc.”. The genotype of each strain is shown in the table below the graphics, where: blanks indicate the WT alleles; Δ indicates disruption of the gene by replacement with a *kan-tdk* marker; for the *kan-tdk* marker line, + indicates the presence and - indicates the absence of the marker; and for the adaptation line, + indicates the presence of adaptation and blanks indicate the absence of adaptation.

Considering that the accumulation of deletions seems to significantly reduce the production of metabolites in the mutant strains, we combined the data from HPLC, NMR, and GC analyses and performed a carbon and electron flux analysis to investigate how carbon and electrons from the substrate source (cellobiose) are used by each strain (Fig. 5). For this analysis, only the strains able to grow in MTC-6 medium were considered. Since we did not quantify cell mass here, the percentage of carbon flux to the cells was not included in our analysis. However, we have observed a 3–10% carbon flux to *T. saccharolyticum* cells in previous work (49, 50). Even taking this into account, it can be noted that many of our strains exhibited <70% carbon recovery, suggesting that their carbon balances are not closed within our experimental accuracy (90%–110%). A common feature of strains with low carbon recovery is the presence of the *hfsD* deletion: Δ*nfnA* Δ*hfsD* (A2G0060), Δ*nfnA* Δ*hydA* Δ*hfsD* (adapted; A2G0071), Δ*nfnAB* Δ*hfsD* (A2G0061), and Δ*nfnAB* Δ*hydA* Δ*hfsD* strains, both before and after adaptation (A2G0063 and A2G0073). This result is consistent with our previous work, which showed that among *nfnA*, *nfnB*, *hydA*, and *hfsD* single knockouts, only the Δ*hfsD* strain exhibited low carbon recovery (19). The electron balance follows the same pattern as the carbon balance; i.e., all strains with the *hfsD* deletion also have the lowest electron recovery (<70%). All other mutant strains, despite having slightly lower carbon and electron fluxes than the LL1328 ethanologenic strain, exhibited near-complete carbon and electron recoveries.

### The growth of the triple and quadruple mutants is impaired by the inhibition of the PFL reaction

**Fig 6:**
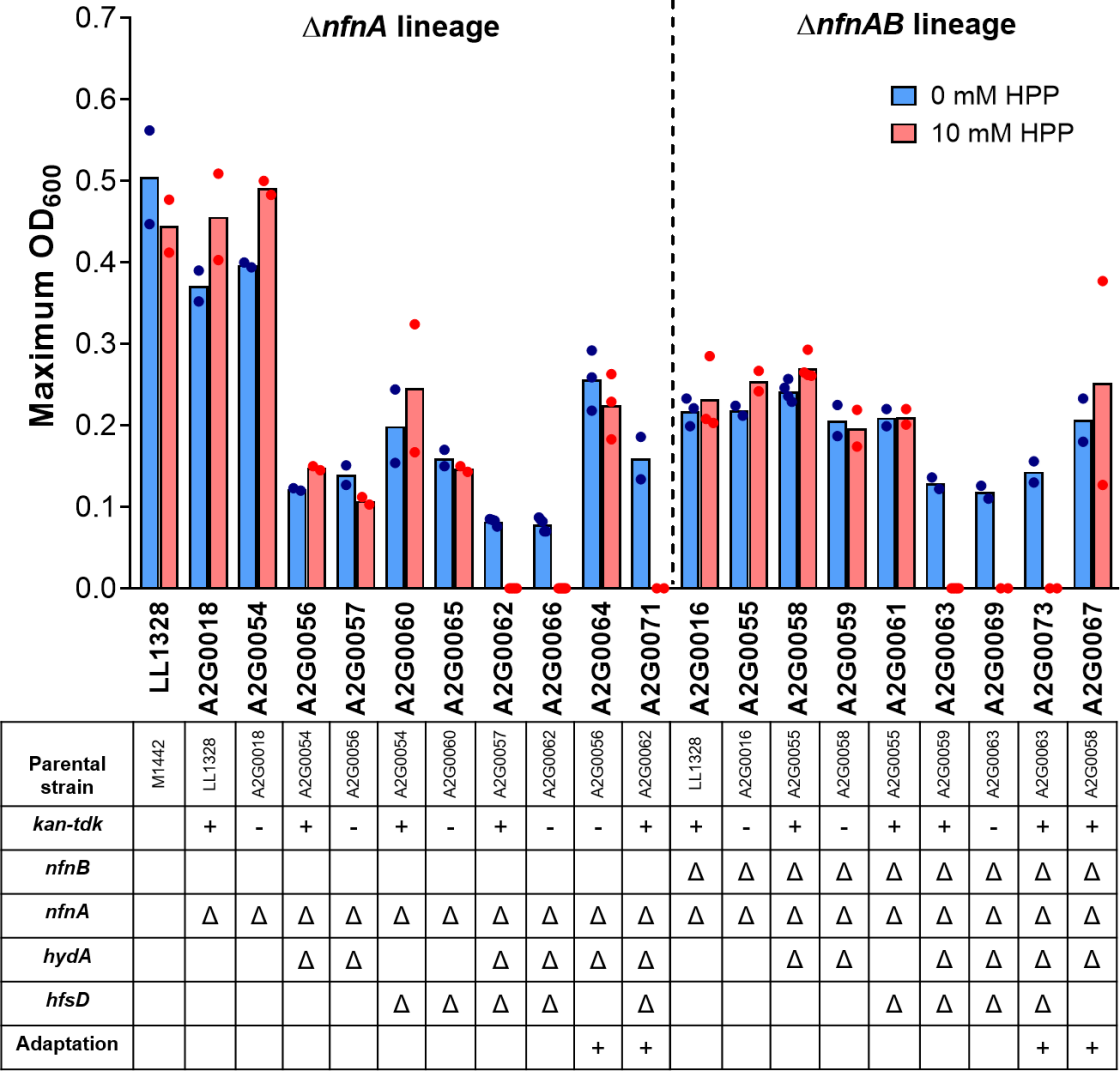
Growth of the strains in the presence of HPP. The strains were cultivated in a 96-well plates containing M122C-rich medium supplemented or not with 10 mM of HPP. Growth was monitored for 96 h at 55°C, and the maximum OD_600_ is plotted. Dots indicate biological replicates. The dashed line separates the strains according to the parental lineage: on the left are the strains derived from Δ*nfnA* lineage, and on the right are the strains derived from Δ*nfnAB* lineage. The genotype of each strain is shown in the table below the graphics, where: blanks indicate the WT alleles; Δ indicates disruption of the gene by replacement with a *kan-tdk* marker; for the *kan-tdk* marker line, + indicates the presence and - indicates the absence of the marker; and for the adaptation line, + indicates the presence of adaptation and blanks indicate the absence of adaptation. Complete growth curves in both media (M122C and MTC-6) are shown in Supporting Fig. S2.

Given the evidence that some mutant strains have begun to divert metabolic flux from Pfor to Pfl, we hypothesized that such strains would be susceptible to sodium hypophosphite (HPP), an analog of formate and known irreversible inhibitor of Pfl (51, 52). Therefore, we tested this experimentally by growing the strains in both rich and defined medium supplemented with different concentrations of HPP (Supporting Fig. S2). Although HPP did not have a significant effect on the growth of the LL1328 parental strain and most mutant strains, even at high concentrations (up to 100 mM), it completely inhibited the growth of the Δ*nfnA* Δ*hydA* Δ*hfsD* and Δ*nfnAB* Δ*hydA* Δ*hfsD* mutant strains, both with or without the *kan* marker (A2G0062, A2G0066, A2G0063, and A2G0069), as well as the Δ*nfnA* Δ*hydA* Δ*hfsD::kan-tdk* and Δ*nfnAB* Δ*hydA* Δ*hfsD::kan-tdk* adapted strains (A2G0071 and A2G0073), at low concentrations (1–10 mM) (Fig. 6; Supporting Fig. S2). To confirm that HPP is capable of inhibiting formate production by the PFL enzyme, we performed a bench fermentation in both the presence and absence of HPP (Supporting Fig. S2). As expected, 10 mM HPP was sufficient to reduce the formate concentration in all tested strains by 60–99%. However, it is worth mentioning that the formate peak in the chromatograms of the samples treated with HPP was difficult to quantify due to the presence of another peak with a similar retention time whose identity could not be determined (Supporting Fig. S3A). In any case, it was clear that HPP decreased formate production. These results show that in our Δ*nfnA* Δ*hydA* Δ*hfsD* and Δ*nfnAB* Δ*hydA* Δ*hfsD* strains, the Pfl enzyme is essential for growth, providing additional evidence that we have diverted flux away from the Pfor enzyme.

### Spontaneous mutations after adaptive evolution

In this work, we started with a strain with a balanced but relatively constrained metabolism (strain LL1328). We then disrupted its electron transfer pathways. In many cases, this eliminated growth in defined medium; however, growth on rich medium (which, due to the presence of additional nutrients, provides the organism with more flexibility for maintaining redox balance) was still possible. We then attempted to adapt the strains for growth in defined medium by serial transfer with gradually decreasing concentrations of rich medium and finally performed whole-genome sequencing to identify mutations that may have allowed the strains to compensate for the targeted mutations.

Spontaneous mutations in the DNA sequence were found in the genome of all the adapted strains (Supporting Table S5). Mutations occurred in genes involved in various processes, such as DNA and RNA replication, ATP metabolism, membrane transport, and others. But surprisingly, three specific genes were most frequently mutated: the transcriptional regulator GntR, the phosphate uptake regulator PhoU, and the alcohol dehydrogenase AdhA (Table 3). Two other genes with potentially interesting mutations include *hfsD* and Tsac_1921 (inorganic polyphosphate/ATP-NAD kinase) (Supporting Table S5).

**TABLE 3:** Most frequent spontaneous mutations in *T. saccharolyticum* mutant strains after adaptive evolution*^b^*^,*c*,*d*^

			Adapted strain (colony)										
			A2G0064 ∆*nfnA* ∆*hydA*		A2G0071 ∆*nfnA* ∆*hydA* ∆*hfsD::kan-tdk*		A2G0067 ∆*nfnAB* ∆*hydA::kan-tdk*				A2G0073 ∆*nfnAB* ∆*hydA* ∆*hfsD::kan-tdk*		
Locus	Gene	Locus description	1	2	1	2	1	2	3	4	1	2	3
Tsac_2087	*adhA*	Alcohol dehydrogenase	G50D		G50D	G50D	G50D	G50D, G81A	G50D	G50D	G50D	G50D, G81A	G50D, G81A
Tsac_0749	*gntR*	GntR family transcriptional regulator		L110	A24T	A24T			R53S	R53S	L36P	N23	K151
Tsac_1588	*phoU*	Phosphate uptake regulator					Stop 219^*a*^			N200	N200		M171

^*a*^ Stop codon was substituted by another amino acid, generating a readthrough protein.

^*b*^ All other mutations found are reported in Supporting Table S5.

^*c*^ Underlined mutations are the deletions that caused frameshift in the protein.

^*d*^ In grey are the insertions that caused frameshift in the protein.

In strains where *hydA* had been deleted, we occasionally observed amino acid substitutions in *hfsD*. In strain A2G0067 (Δ*nfnAB* Δ*hydA::kan-tdk* adap), we observed a Lys53Arg mutation. In strain A2G0064 (Δ*nfnA* Δ*hydA* adap), we observed a Lys98Glu mutation. Although we did not perform any further characterization of these mutations, they may be important for understanding the interaction between the Hfs and Hyd hydrogenases.

In two independent strains from the *nfnA* deletion lineage (strain A2G0064: Δ*nfnA* Δ*hydA* adap and A2G0071: Δ*nfnA* Δ*hydA* Δ*hfsD::kan-tdk* adap), we observed mutations in Tsac_1921 (inorganic polyphosphate/ATP-NAD kinase), which uses a phosphate donor (ATP or polyphosphate) to convert NAD^+^ to NADP^+^. One mutation was a frameshift, while the other was silent. Although not widespread among the strains, its putative function in balancing NAD^+^ and NADP^+^ redox pools suggests a possible mechanism of adaptation to the redox imbalance imposed by the *hydA* deletion.

GntR is a family of transcription factors distributed across various bacteria that activates or represses gene expression in response to environmental stress or other types of signals, such as carbon source availability, impacting different biological processes (53, 54). Although *T. saccharolyticum* has eight genes annotated as GntR family transcriptional regulators, only one (*Tsac_0749*) was mutated. Mutations in the *gntR* gene were observed in at least one replicate of each *T. saccharolyticum* adapted strain, occurring in two different ways: missense single nucleotide variations (SNVs) (A24T, R53S, L36P), as well as single nucleotide deletions in different regions of the genomic sequence, leading to frameshift proteins.

In bacteria, PhoU is a regulator of phosphate homeostasis and its counterions, impacting processes such as cell signaling, transport, and stress response (55). One unique aspect of the *phoU* mutations is that they were only observed in strains derived from the Δ*nfnAB* lineage. The *phoU* mutations were all frameshifts, which would be expected to eliminate activity.

AdhA is the final enzyme in the ethanol production pathway, responsible for converting acetaldehyde into ethanol using NADPH as a cofactor (7). Despite being the most frequently mutated gene, only two specific missense SNVs occurred in *adhA*: a C-to-T transition that culminated in the G50D mutation in the protein sequence of all adapted strains except for one replicate (A2G0064-2), and a C-to-G transversion that culminated in the G81A mutation in the protein sequence of three strains. However, when compared to the genomes of the parental strains, we observed that the G50D mutation originated earlier in the Δ*nfnAB* lineage (NCBI’s Short Read Archives from Bioproject PRJNA1314240). While the strains from the Δ*nfnA* lineage did not present this mutation (only the adapted ones), the AdhA G50D mutation was found in the genome of three strains from the Δ*nfnAB* lineage that did not undergo adaptive evolution: the Δ*nfnAB* Δ*hydA* strain without *kan* marker (A2G0059) and the Δ*nfnAB* Δ*hydA* Δ*hfsD* strains both with and withou*t kan* marker (A2G0063 and A2G0069, respectively). Therefore, it is correct to infer that the G50D mutation of the Δ*nfnAB* Δ*hydA* Δ*hfsD::kan-tdk* adapted strain (A2G0073) was inherited from the parental A2G0063 strain (which in turn came from the A2G0059 strain). But the same did not happen with the Δ*nfnAB* Δ*hydA::kan-tdk* adapted strain (A2G0067), since it is derived from strain A2G0058, which does not have the mutation. Considering this result and the growth analysis in Fig. 2, it is possible to conclude that the chain of gene knockouts led to growth impairment in defined medium in both lineages (Δ*nfnA* and Δ*nfnAB*), but interestingly, all the strains that managed to recover growth developed the AdhA G50D spontaneous mutation, either naturally (strains A2G0059, A2G0063, A2G0069, and A2G0073) or during adaptive evolution (strains A2G0064, A2G0071, and A2G0067). In other words, we can hypothesize that the G50D mutation is enabling the growth of these strains.

### Characterization of the spontaneous mutations

In order to better understand the reason for the appearance of these spontaneous mutations, we reintroduced the G50D mutation of AdhA (the most common) into the LL1328 homoethanologenic strain and completely deleted the *gntR* and *phoU* genes, both also in the LL1328 strain. We chose to delete *gntR* and *phoU* due to the numerous single nucleotide deletions/insertions found in these genes that affected the reading frame of the respective proteins, something that can be compared to and better investigated with a knockout.

Both the *gntR* and *phoU* deletions had minimal impact on fermentation and growth (Supporting Fig. S4). While the *gntR* knockout did not significantly change the fermentation profile of the LL1328 strain, the *phoU* knockout slightly decreased the levels of ethanol and acetate and increased formate levels. Both strains grew the same way as the LL1328 strain in M122C-rich medium; however, they reached lower maximum OD values in MTC-6 defined medium, exhibiting a small growth defect.

**Fig 7:**
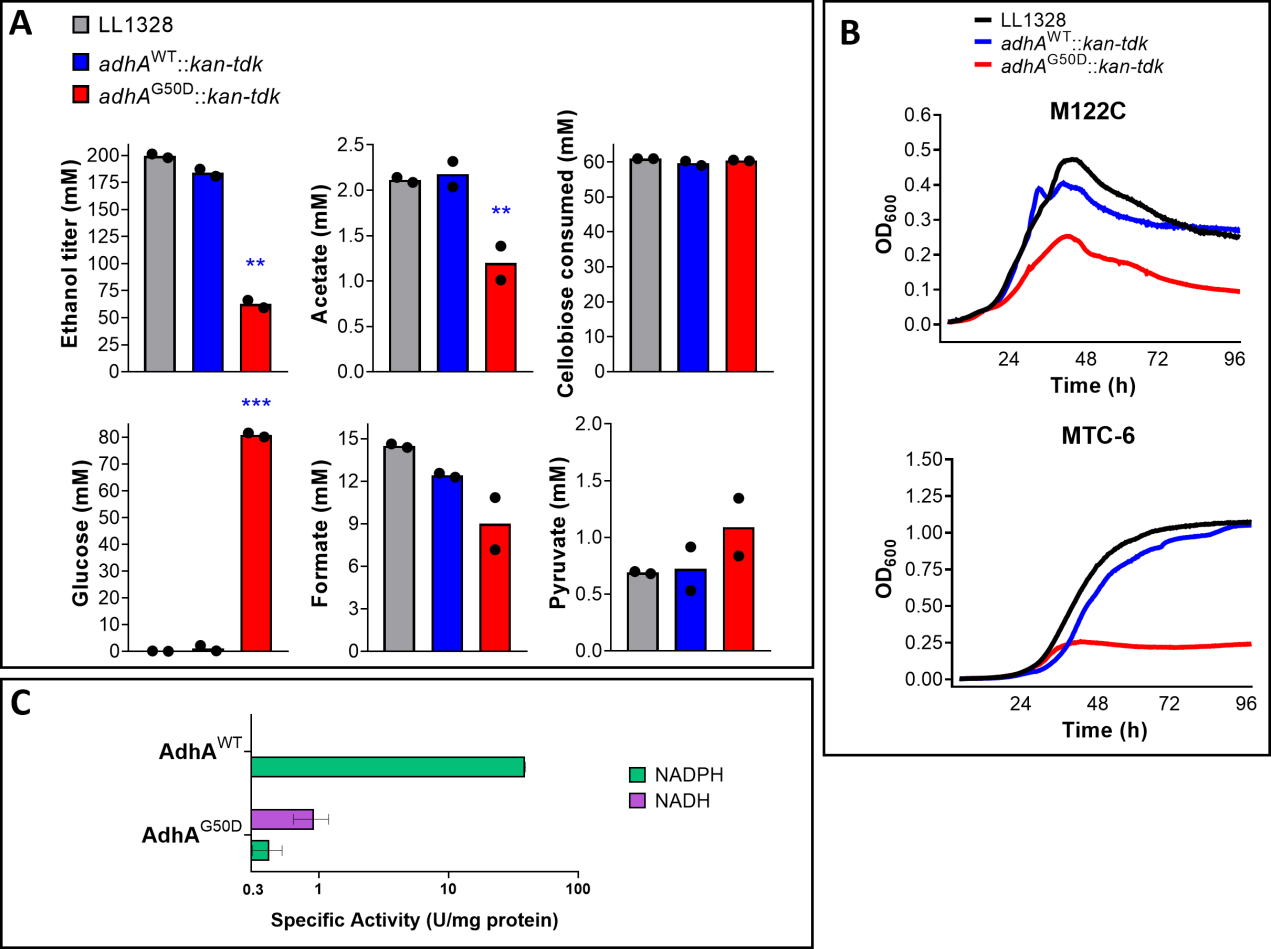
Characterization of the AdhA G50D mutation. (**A**) Fermentation profile of the strains. All the strains were cultivated on MTC-6 defined medium containing 20 g/L of cellobiose for 7 days. Black dots indicate the biological replicates. ** *P* ≤ 0.01 and ***P ≤ 0.001 (One-way ANOVA and Dunnett’s post-test in relation to LL1328 strain—light gray bar). (**B**) Growth phenotype of the strains. For the growth analysis, the strains were cultivated in a 96-well plates containing MTC-6 medium or M122C medium for 96 h at 55°C. Readings were taken every 8 min. The strain containing the *adhA*^WT^::*kan-dk* cassette (blue bar and curve) was used as a control for the effect of the *kan* gene. (**C**) Enzymatic activity of wild-type and G50D AdhA enzymes. Each enzyme was heterologous expressed in *E. coli*, purified, and assayed anaerobically at 55°C in the physiological direction (acetaldehyde + NAD(P)H → ethanol + NAD(P)^+^), using either NADH or NADPH as cofactors.

As for the AdhA G50D mutation, both fermentation and growth were strongly impacted by the amino acid change (Fig. 7A and B). Ethanol titer decreased approximately 70% compared to the LL1328 parental strain, and acetate levels decreased 40%. Although the G50D mutant strain consumed all the cellobiose in the medium, it accumulated high levels of glucose, a phenotype similar to mutants with *nfnA* or *nfnAB* deletion (Fig. 4 and 5).

Due to the proximity of the G50 residue to the NADPH binding site, we hypothesized that the G50D mutation might affect cofactor specificity for NADPH vs NADH. To test this hypothesis, we heterologously expressed and purified both proteins, wild-type AdhA and G50D AdhA (Supporting Fig. S5), and measured their activity in the presence of NADPH or NADH (Fig. 7C). As expected, the wild-type enzyme had almost exclusively NADPH-linked ADH activity (the NADH activity was below the detection threshold). The mutated protein, however, lost more than 98% of its NADPH-linked ADH activity and acquired NADH-linked ADH activity (although with levels lower than the NADPH activity that was lost). Taken together, these results suggest that the combined deletion of Fnor and hydrogenase enzymes led to a cellular disturbance in which NADPH production was decreased, resulting in decreased ethanol production due to the strict NADPH-linked ADH activity of AdhA. The mutations in AdhA may have served several purposes: (i) increasing the availability of NADPH for biosynthesis by avoiding NADPH consumption for ethanol production, (ii) allowing detoxification of acetaldehyde by conversion to ethanol using NADH, and/or (iii) providing an additional sink for NADH oxidation.

### Structural effects of AdhA and GntR point mutations

**Fig 8:**
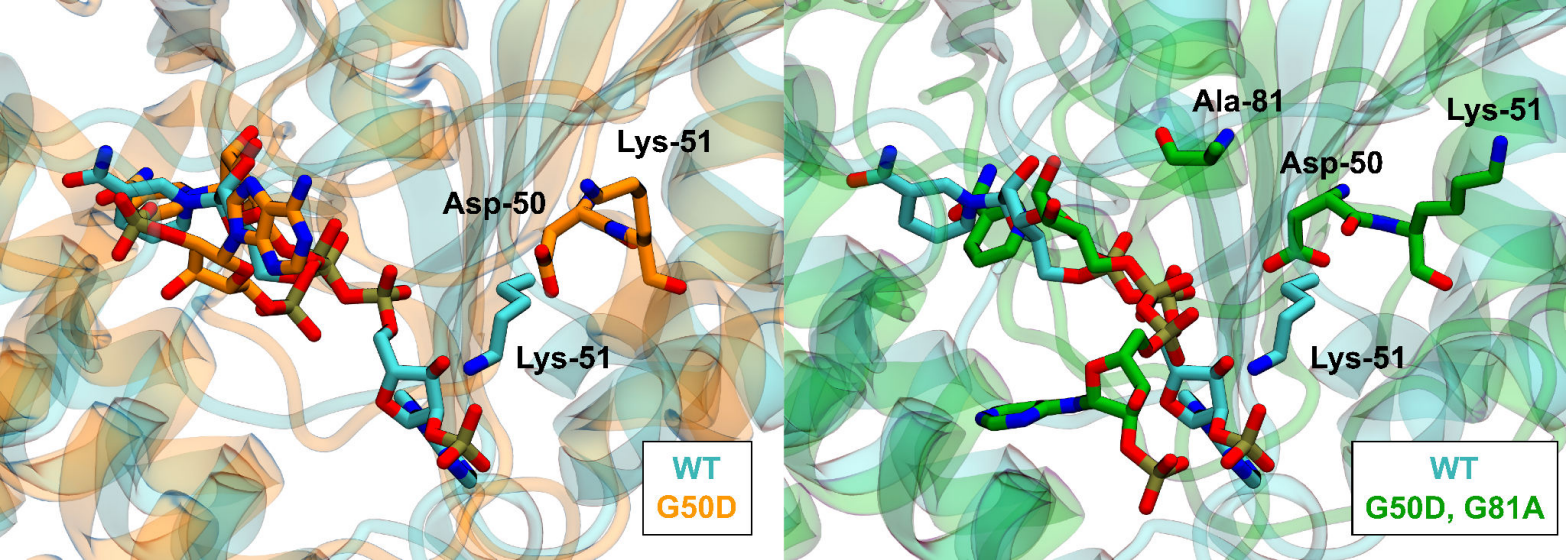
AdhA structural dynamics mutation effects. Snapshots from molecular dynamics simulations of wild-type (WT, teal), G50D (orange), and G50D, G81A (green) variants of AdhA. Both mutations have the effect of tending to rotate the sidechain of Lys-51 away from its native role in stabilizing the phosphate groups of the NADPH cofactor. The WT Lys-51 is shown interacting with the 2′-phosphate of NADPH, whose interaction is disrupted in both mutants. See Supporting Fig. S6 for data quantifying this effect. The G50D and G81A mutations were modeled together because they co-occurred in the strains in which they appeared.

To investigate the effects of the identified point mutations on AdhA (G50D and G81A) and GntR (A24T, R53S, L36P) proteins, we performed molecular dynamics simulations. *T. saccharolyticum* AdhA is a 400-amino-acid protein, of which most make up an iron-containing alcohol dehydrogenase (ADH) domain (22). Both spontaneous mutations found in this protein are inside the ADH domain. Because of the proximity of the mutations to the cofactor binding site, we hypothesized that they might interfere with cofactor binding or recognition. Although the cofactor generally remained bound over the course of our simulations, significant disruptions in the stabilization of its conformation were observed as a consequence of the mutations. In particular, the residue Lys-51 was observed to play a major role in stabilizing the negatively charged phosphate groups on NADPH (both the pyrophosphate group and the 2′-phosphate), but both G50D and G81A mutations altered the positioning of that residue to inhibit these interactions, as shown in Fig. 8. Of the seven positively charged residues (all of them lysines) on the surface of the protein near the pocket occupied by the 2′-phosphate in the NADPH-bound conformation, only one other—Lys-199—was ever observed to directly interact with it during our simulations of the wild-type protein and did so with less than one fifth the frequency of Lys-51, emphasizing the importance of the latter in coordinating the cofactor. Other than those residues, the 2′-phosphate interacts mostly only with the solvent.

**Fig 9:**
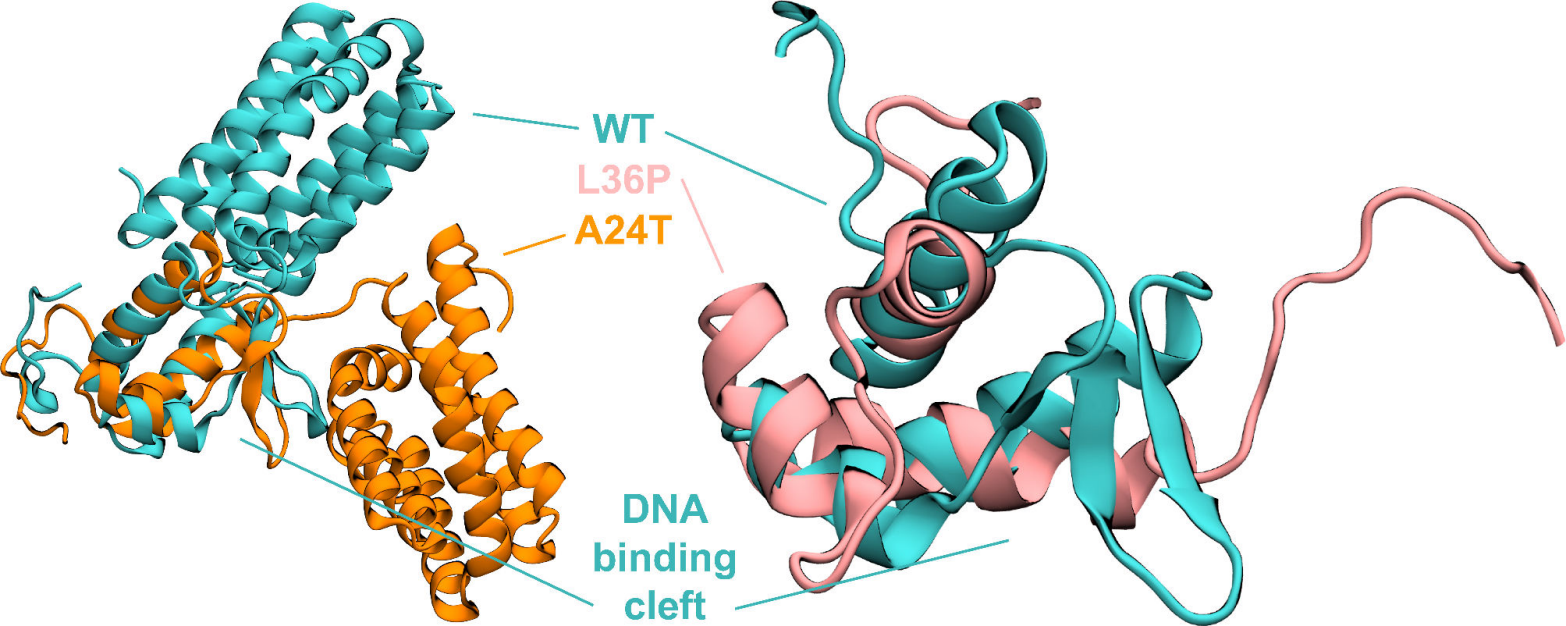
GntR structural dynamics mutation effects. Snapshots from molecular dynamics simulations of wild-type (WT, teal), L36P (pink), and A24T (orange) variants of GntR transcriptional regulator. A24T encourages a “closed” conformation between the DNA-binding and effector domains, likely interfering with DNA binding. See Supporting Fig. S7 for data quantifying this effect. L36P directly unfolds key portions of the DNA-binding cleft in the DNA-binding domain.

*T. saccharolyticum* GntR (Tsac_0749), a protein with 226 amino acids, shares 87% similarity and 29% identity (e-value 1e-15) to UxuR, an *E. coli* GntR transcription regulator from the FadR subfamily (53). The proteins of this family share a highly similar N-terminal HTH (helix-turn-helix) DNA-binding domain. Here, all the point mutations observed in the *gntR* gene were inside its DNA-binding domain. In our simulations, each mutation had distinctive effects, but all appeared likely to interfere with the binding of the DNA-binding domain to DNA in different ways. The most straightforward of these is R53S: Arg-53 extends into the binding site directly and should be responsible for recognizing a negative charge on the target DNA molecule, whereas serine is uncharged and cannot reach as deeply into the major groove of the helix. Even more disruptively, L36P completely unfolds the local structure of the DNA-binding domain. Most subtly, A24T appears at first to be unimportant, but upon simulation was observed to greatly alter the interdomain dynamics, causing the effector domain to favor a “closed” conformation that bends it into the path that the DNA helix would occupy. These latter two effects are visualized in Fig. 9. These observations, alongside the frequency with which frameshift mutations were observed in GntR, support the hypothesis that adaptive evolution favored disruptions to the function of this protein, which may result in changes in the expression levels of genes that it regulates.

Despite the likely inactivation of GntR during adaptive evolution experiments, deletion of this gene did not result in a clear phenotype in the homethanologenic strain (Supporting Fig. S4). However, besides GntR being known as a negative regulator of the gluconate operon, studies have shown that some variants of this protein also regulate genes involved in glucose uptake/transport and the pentose phosphate pathway (56–59). Considering that this metabolic route is an alternative NADPH-generating pathway (60), mutations in the GntR regulator may be an evolutionary adaptation of the mutant strains of this study to cope with the excessive accumulation of unmetabolized glucose and the lack of NADPH. However, the exact GntR target genes affected by these structural changes and their relationship to redox imbalance still need to be elucidated.

## DISCUSSION

The intracellular transfer of electrons that occurs during the fermentation of anaerobic bacteria moves electrons in a coordinated manner from ferredoxin to nicotinamide cofactors to organic products such as lactate, acetate, or ethanol. Understanding this process allows for the optimization of the microorganism’s energy metabolism, as well as the development of strategies to increase the yield of these final organic electron acceptors. Not surprisingly, genetic manipulations that directly affect electron flux significantly impact carbon flux and ethanol production (61–63).

Previously, we observed that the Fnor enzymes NfnA and NfnB and the hydrogenases HydA and HfsD are necessary for the homoethanologenic phenotype of a *T. saccharolyticum* strain (19). Our hypothesis is that the four enzymes act together to promote electron transfer from ferredoxin to NADPH via hydrogen cycling. Based on homology to other characterized hydrogenases, we predict that HfsD is monofunctional, and HydA is part of the HydABC holoenzyme, which acts as a bifurcating hydrogenase (64). With the HfsD hydrogenase operating in the hydrogen-forming direction and the Hyd hydrogenase operating in the hydrogen-consuming direction (Fig. 1), a net transfer of electrons from Fd_red_ to NAD^+^ can be achieved (equivalent to a NADH-linked FNOR reaction). This work further supports the hydrogen cycling hypothesis by demonstrating that NfnA, NfnB, HfsD, and HydA are the primary enzymes associated with oxidation of the Fd_red_ generated by the Pfor enzyme, as shown by PFOR/PFL reaction flux calculations and measurement of hydrogen production (Fig. 3). However, detailed biochemical validation of hydrogen cycling is still needed.

One remaining question is where hydrogen comes from in the strains where *hfsD* and *hydA* have both been deleted. One possibility is that the remaining Ech hydrogenase (48) exhibits low levels of activity. Another possibility is that the remaining subunits of the Hfs hydrogenase (HfsABC) can form a functional hydrogenase in the absence of HfsD. Support for this hypothesis is found in work on *Ruminococcus albus* hydrogenases, where genes corresponding to the *hfsABCD* operon from *T. saccharolyticum* were described as separate hydrogenases HydS (corresponding to HfsB) and HydA2 (corresponding to HfsD) (64).

In our prior work, we observed that the NfnB protein could function in the absence of NfnA (19). In this work, we were interested to understand whether NfnB could function as the sole ferredoxin oxidation enzyme. Based on the inability of the ∆*nfnA* ∆*hydA* ∆*hfsD* adap strain (A2G0071) to restore the high ethanol yield phenotype, even after adaptation by serial transfer, we provide evidence that NfnB does not function as a “true” Fnor (65) under physiological conditions. Interestingly, in many cases, the *nfnA* deletion strains (i.e., with a functional *nfnB*) had more severe growth defects than the *nfnAB* deletion strains (Fig. 2 and 3). But our data suggest that this phenotype is due to the AdhA G50D mutation, which, by appearing spontaneously, enabled the growth and ethanol production of strains derived from the ∆*nfnAB* lineage.

With respect to metabolism, while the deletion of *nfnA* or *nfnAB* led to an accumulation of acetaldehyde due to a lack of NADPH needed for its further conversion to ethanol, the subsequent deletion of *hydA* and/or *hfsD* decreased the supply of NADH, limiting the pathway at the NADH-linked ALDH reaction and causing an accumulation of upstream metabolites (pyruvate, formate, and acetoin) (Fig. 4). However, the reasons why such deletions and consequent redox imbalance cause the accumulation of glucose, and the identity of the unmeasured fermentation products that account for the remaining percentage of carbon and electron balances (Fig. 5), still require further investigation.

The experiments we have shown in this work provide a much clearer picture of electron transfer in *T. saccharolyticum*. During the initial work to engineer *T. saccharolyticum* for high-yield ethanol production, the *ldh* and *pta-ack* genes were deleted to eliminate lactate and acetate production (21, 66). The strain rapidly adapted to these constraints by disrupting the *hfsB/C* genes or introducing point mutations in the *hfsD* gene (13, 67). Although the exact mechanism of these mutations is still not known, they appear to have enabled the net transfer of electrons from Fd_red_ to NAD^+^ via hydrogen cycling (19, 20). This, in turn, allowed strains with WT *adhE* genes to produce ethanol at high yield (>80% of theoretical maximum) (24).

Adaptation of high ethanol-yielding strains of *T. saccharolyticum* for increased ethanol tolerance (as well as tolerance to hardwood hydrolysate) resulted in mutations to the *adhE* gene (13, 67). Initially, it was hypothesized that these mutations improved the affinity of AdhE for acetyl-CoA or NADPH; however, subsequent reintroduction of those mutations did not have a significant effect on ethanol production (67). Following analysis of these mutations determined that their effect was to eliminate activity of the ADH domain of the AdhE enzyme, which in turn allowed increased ethanol tolerance (68). In this strain, the lack of NADH-linked ADH activity did not cause a decrease in ethanol production due to the presence of the NADPH-linked AdhA enzyme, which took over responsibility as the primary ADH enzyme (22). The NADPH demand from the AdhA enzyme, in turn, required function of the NfnAB complex. In engineered *T. saccharolyticum* strains that rely on AdhA (e.g. M1442), the NfnAB protein complex is essential for high-yield ethanol production (24).

Having engineered a strain of *T. saccharolyticum* with primarily NADPH-linked ADH activity (via AdhA), we now eliminated all of the electron transfer pathways from ferredoxin by deleting *nfnAB*, *hfsD*, and/or *hydA*, largely blocking flux through the PFOR reaction (Fig. 3). These strains responded by producing acetaldehyde, formate, and/or pyruvate (Fig. 4), suggesting an accumulation of NADH. This provided selective pressure for the NADPH-linked AdhA gene to acquire mutations giving it some NADH-linked activity (Fig. 6 and 7).

In addition, one useful application of the strains developed in this work is as a platform for screening candidate Fnor enzymes. Our group has recently screened 27 Fnor enzymes from different organisms and found four promising candidates with high thermostability and “true” FNOR activity (65). We have shown that these enzymes can be used to produce ethanol *in vitro* using purified enzymes, but so far we have not been able to assemble them into an *in vivo* high-yield and titer ethanol production pathway. The cumulative deletion strains developed in this work (Δ*nfnAB* Δ*hydA* Δ*hfsD*) may be useful for understanding the cellular context necessary for heterologous Fnor enzymes to function efficiently in ethanol production pathways.

In conclusion, this work showed that Fnors and hydrogenase enzymes work in an integrated way to ensure intracellular redox balance and control the availability of reduced electron carriers for the ethanol production pathway of *T. saccharolyticum*. We provided evidence that disruption of these enzymes leads to a considerable growth defect, redistribution of fermentation products, increased metabolic flux through Pfl, and molecular adaptations to cope with the decreased pool of NADPH. Our data have enhanced the understanding of electron transfer pathways in *T. saccharolyticum* and may assist future efforts to transfer the robust ethanol production pathway from this thermophile to other organisms, with promising applicability in sustainable energy production.

## SUPPLEMENTAL MATERIAL

Supplemental figures jb.00057-26-s0001.pdf Fig. S1 to S7.

Table S1 jb.00057-26-s0002.xlsx Primers used for strains construction.

Table S2 jb.00057-26-s0003.xlsx HPLC data.

Table S3 jb.00057-26-s0004.xlsx GC data.

Table S4 jb.00057-26-s0005.xlsx NMR data.

Table S5 jb.00057-26-s0006.xlsx Spontaneous mutations detected after whole genome sequencing.

Table S6 jb.00057-26-s0007.xlsx Stoichiometry calculations of each reaction in the glycolysis and ethanol production pathway of T. saccharolyticum ethanologenic strain LL1328.

ASM does not own the copyrights to Supplemental Material that may be linked to, or accessed through, an article. The authors have granted ASM a non-exclusive, world-wide license to publish the Supplemental Material files. Please contact the corresponding author directly for reuse.
