# Supplementary material for: Adaptive evolution of electron transfer pathways in Thermoanaerobacterium saccharolyticum
Source: J Bacteriol. 2026 May 28;208(6):e00057-26. doi: 10.1128/jb.00057-26 (PMC13277313; doi:10.1128/jb.00057-26)
Supplement: Supplemental figures — Fig. S1 to S7. [file jb.00057-26-s0001.pdf]

Supporting Information

Supporting Table S1. Primers used for *T. saccharolyticum* transformation and validation.

Supporting Table S2. Batch fermentation data measurements.

Supporting Table S3. Molecular hydrogen measurements.

Supporting Table S4. Nuclear magnetic resonance data.

Supporting Table S5. Secondary mutations in *T. saccharolyticum* adapted mutant strains.

Supporting Table S6. Stoichiometry of each reaction in the pathway.

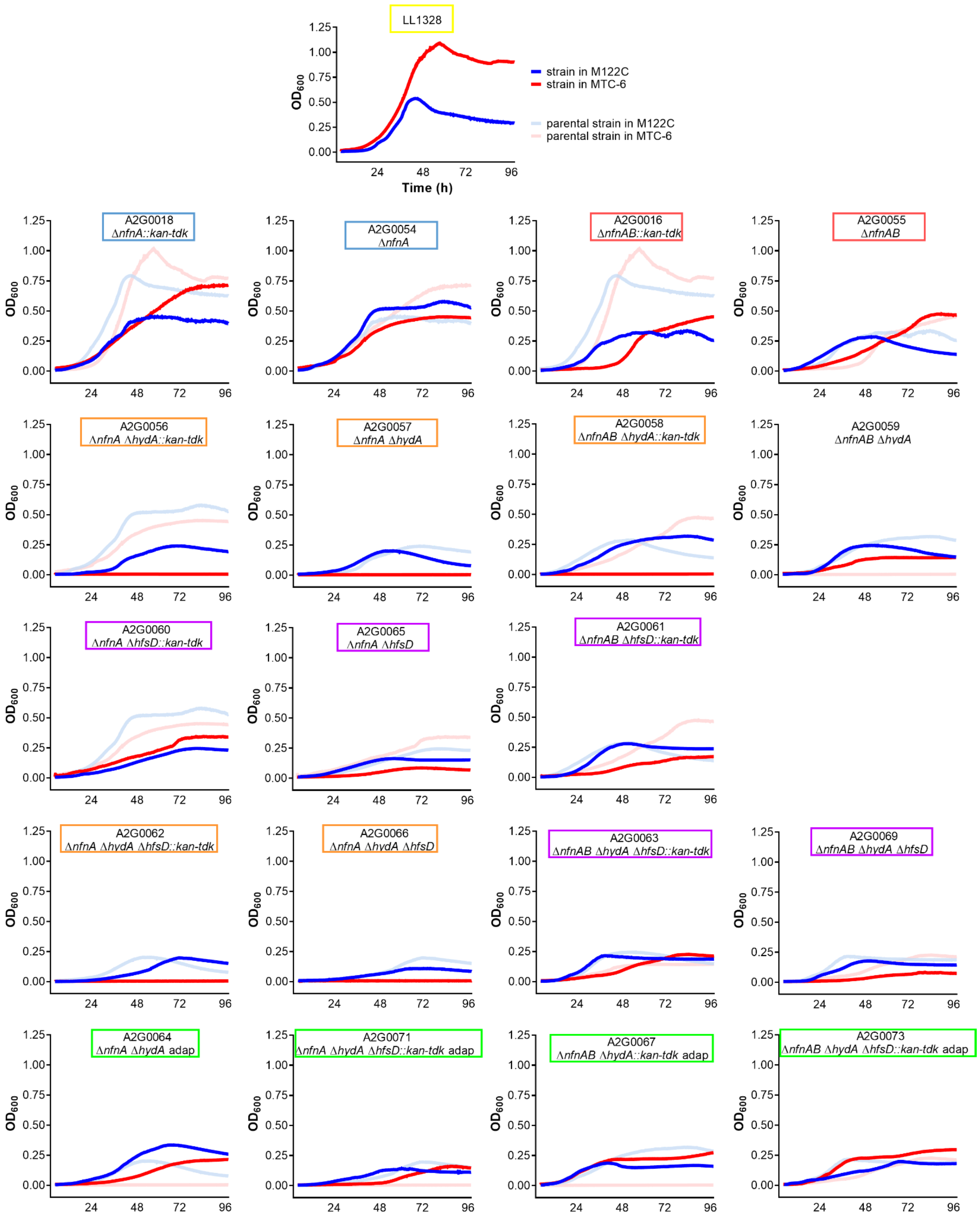

Supporting Figure S1. Growth curves of the strains. For the growth analysis, the strains were cultivated in a 96-well plate containing MTC-6 medium or M122C medium for 96 h at 55 ºC. Readings were taken every 8 minutes. Each curve is representative (of at least two replicates) of the growth of the respective strain in MTC-6 defined medium (dark red) or M122C rich medium (dark blue) along with its parental strain (light red or light blue) in both media. Colored boxes around the strain names match the colors in Figure 1B. For details on the parental strains of each mutant, see Table 1 and Figure 1.

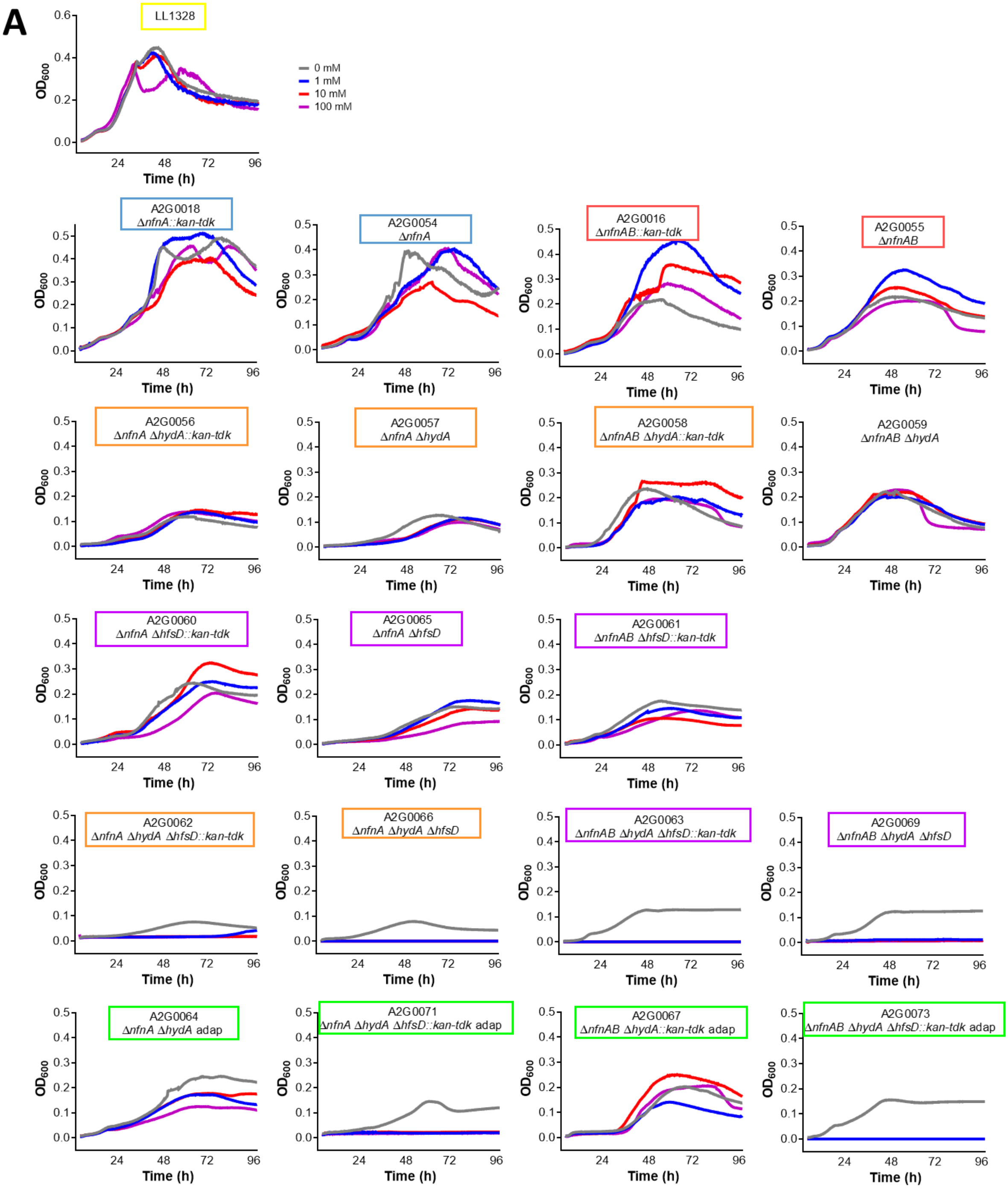
A
LL1328
0 mM
1 mM
10 mM
100 mM
OD600
Time (h)
A2G0018
ΔnfnA::kan-tdk
A2G0054
ΔnfnA
A2G0016
ΔnfnAB::kan-tdk
A2G0055
ΔnfnAB
A2G0056
ΔnfnA ΔhydA::kan-tdk
A2G0057
ΔnfnA ΔhydA
A2G0058
ΔnfnAB ΔhydA::kan-tdk
A2G0059
ΔnfnAB ΔhydA
A2G0060
ΔnfnA ΔhfsD::kan-tdk
A2G0065
ΔnfnA ΔhfsD
A2G0061
ΔnfnAB ΔhfsD::kan-tdk
A2G0062
ΔnfnA ΔhydA ΔhfsD::kan-tdk
A2G0066
ΔnfnA ΔhydA ΔhfsD
A2G0063
ΔnfnAB ΔhydA ΔhfsD::kan-tdk
A2G0069
ΔnfnAB ΔhydA ΔhfsD
A2G0064
ΔnfnA ΔhydA adap
A2G0071
ΔnfnA ΔhydA ΔhfsD::kan-tdk adap
A2G0067
ΔnfnAB ΔhydA::kan-tdk adap
A2G0073
ΔnfnAB ΔhydA ΔhfsD::kan-tdk adap

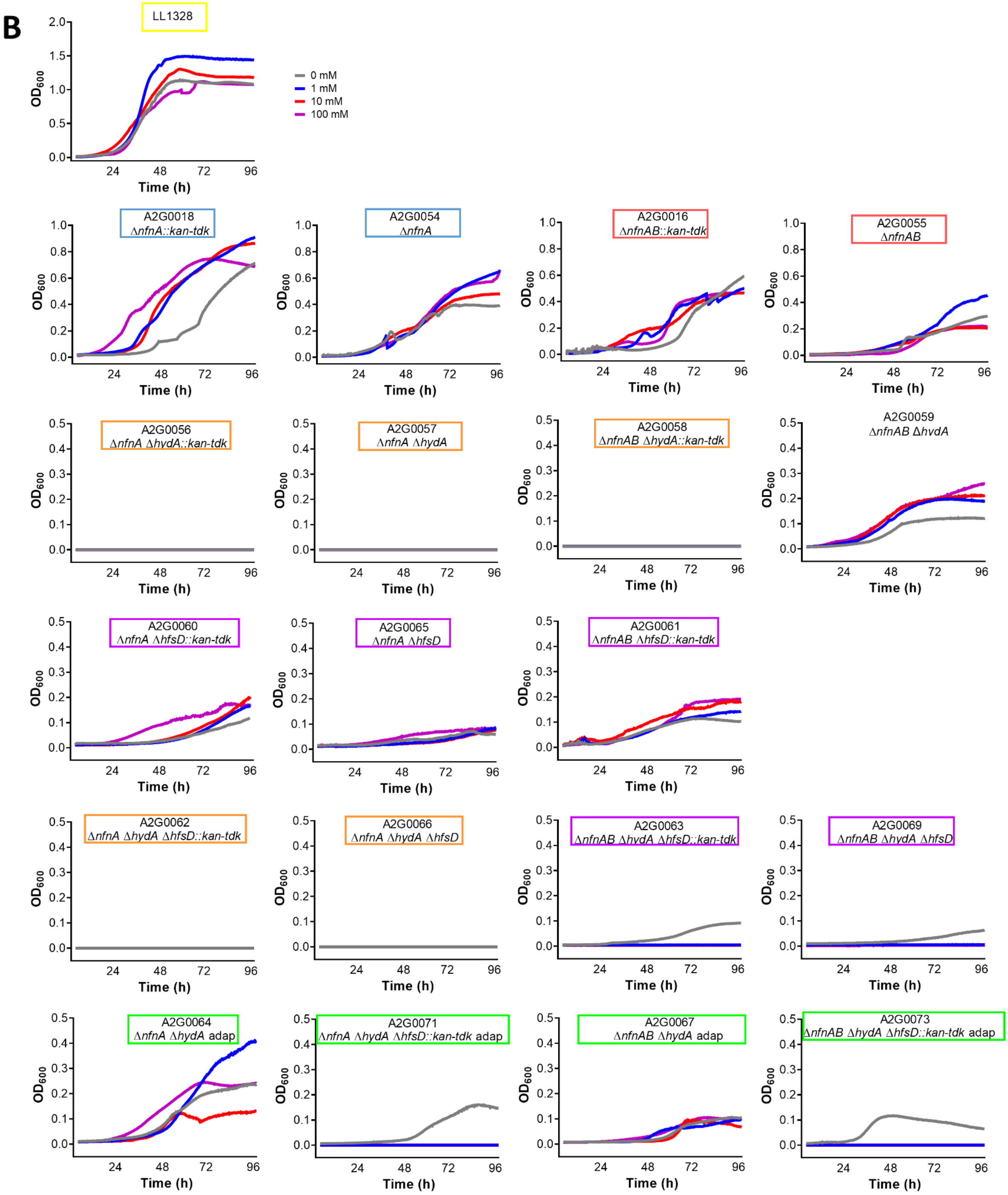

Supporting Figure S2. Growth curves of the strains in different concentrations of HPP. For the growth analysis, the strains were cultivated for 96 h at 55 ºC in a 96-well plate containing M122C medium (A) or MTC-6 medium (B) supplemented with HPP (0, 1, 10 or 100 mM). Readings were taken every 8 minutes. Each curve is representative (of at least two replicates) of the growth of the respective strain in each condition. Colored boxes around the strain names match the colors in Figure 1B.

A

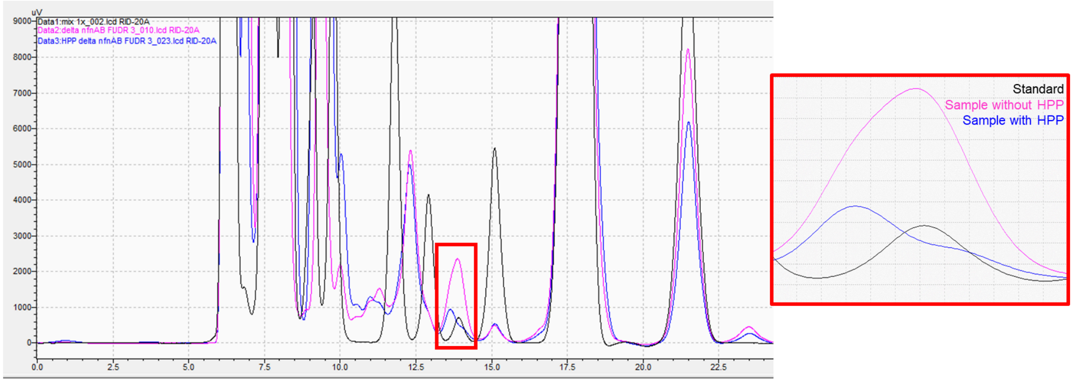

B

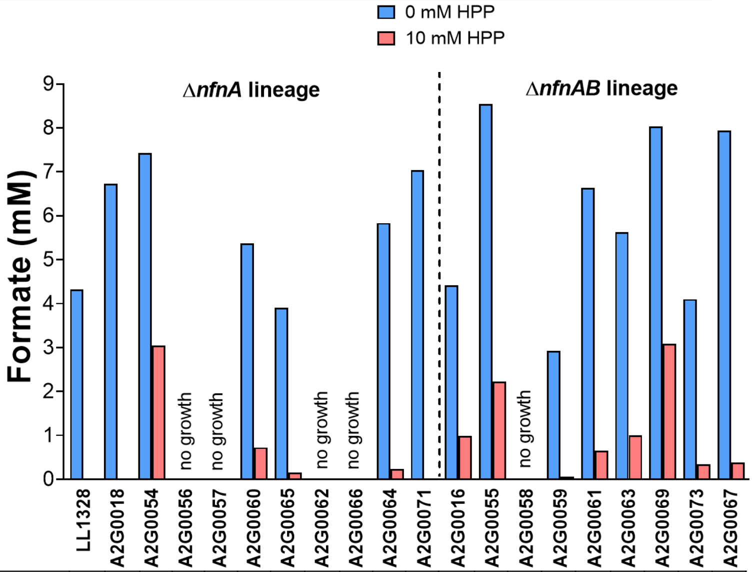

	LL1328	A2G0018	A2G0054	A2G0056	A2G0057	A2G0060	A2G0065	A2G0062	A2G0066	A2G0064	A2G0071	A2G0016	A2G0055	A2G0058	A2G0059	A2G0061	A2G0063	A2G0069	A2G0073	A2G0067
**Parental strain**	M1442	LL1328	A2G0018	A2G0054	A2G0056	A2G0054	A2G0060	A2G0057	A2G0062	A2G0057	A2G0062	LL1328	A2G0016	A2G0055	A2G0058	A2G0055	A2G0059	A2G0063	A2G0063	A2G0058
kan-tdk		+	-	+	-	+	-	+	-	-	+	+	-	+	-	+	+	-	+	+
nfnB												Δ	Δ	Δ	Δ	Δ	Δ	Δ	Δ	Δ
nfnA		Δ	Δ	Δ	Δ	Δ	Δ	Δ	Δ	Δ	Δ	Δ	Δ	Δ	Δ	Δ	Δ	Δ	Δ	Δ
hydA				Δ	Δ			Δ	Δ	Δ	Δ			Δ	Δ		Δ	Δ	Δ	Δ
hfsD						Δ	Δ	Δ	Δ		Δ					Δ	Δ	Δ	Δ	
**Adaptation**										+	+								+	+

Supporting Figure S3. Formate production in the presence of HPP. (A) HPLC chromatogram highlighting the retention time of formate. Black curve: standard; pink curve: sample without HPP; blue curve: sample with HPP. (B) Formate quantification. All the strains were cultivated on MTC-6 defined medium containing 20 g/L of cellobiose for 7 days. The dashed line separates the strains according to the parental lineage: on the left are the strains derived from Δ*nfnA* lineage and on the right are the strains derived from Δ*nfnAB* lineage. The genotype of each strain is shown in the table below the graphics, where blanks indicate the WT alleles and Δ indicates disruption of the gene by replacement with a *kan-tdk* marker.

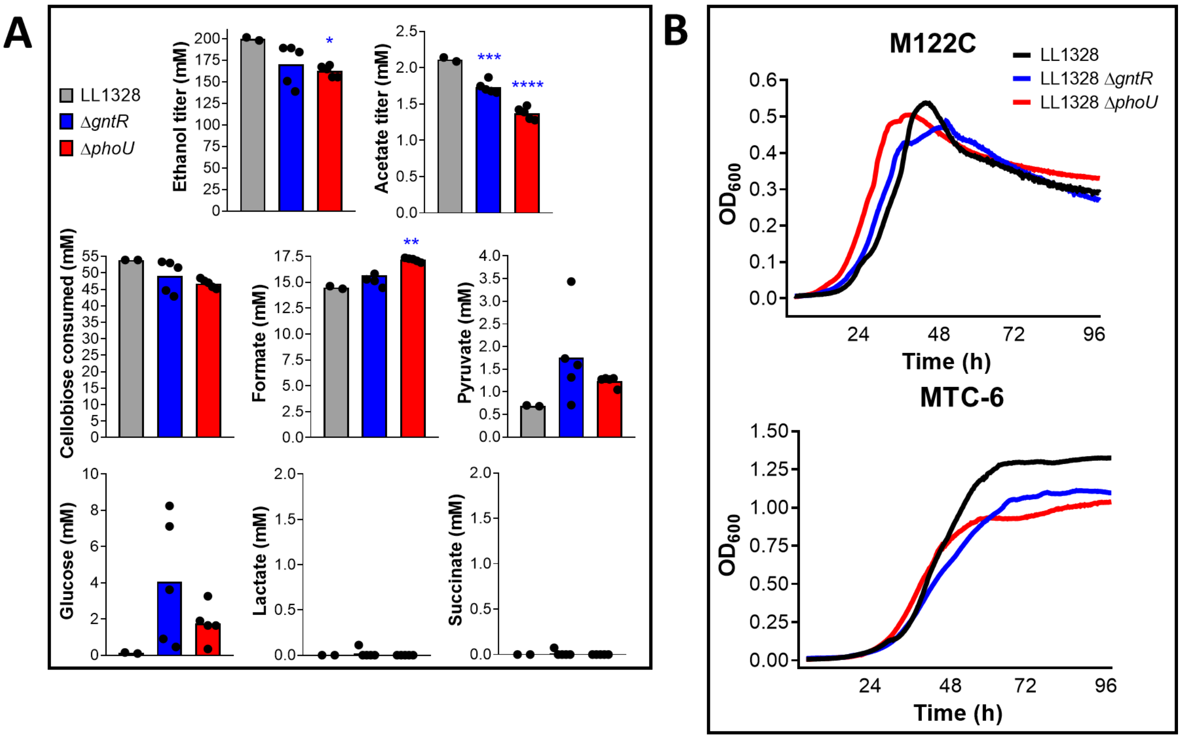

Supporting Figure S4. Characterization of strains containing *gntR* and *phoU* deletions. (A) Fermentation profile of the strains. All the strains were cultivated on MTC-6 defined medium containing 20 g/L of cellobiose for 7 days. Black dots indicate the biological replicates. * $p \leq 0.05$, ** $p \leq 0.01$, *** $p \leq 0.001$ and **** $p \leq 0.0001$ (One-way ANOVA and Dunnet's post-test in relation to LL1328 strain - light grey bar). (B) Growth phenotype of the strains. For the growth analysis, the strains were cultivated in a 96-well plate containing MTC-6 medium or M122C medium for 96 h at 55 ºC. Readings were taken every 8 minutes.

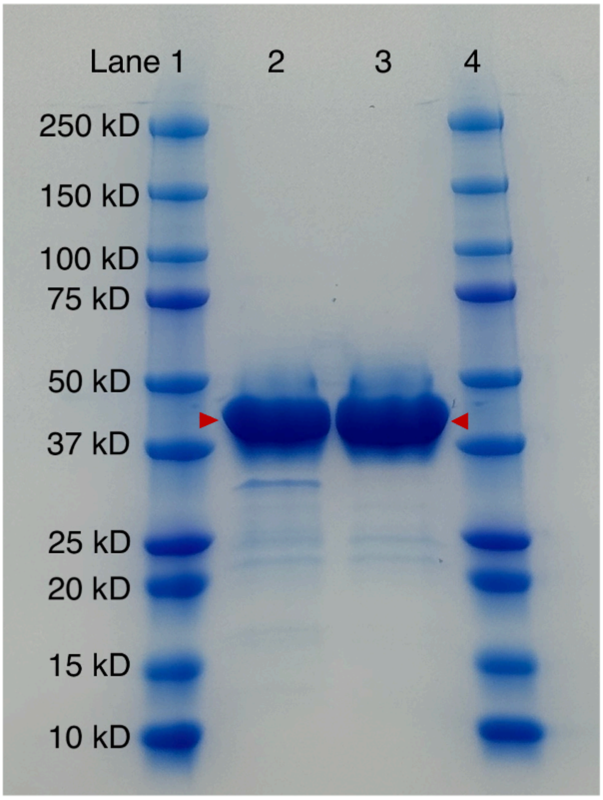

Supporting Figure S5. SDS-PAGE of His-Trap-purified, concentrated AdhA variants. Lanes 1 and 4: Precision Plus Protein Dual-Color Standards. Lane 2: AdhA WT (45 kDa). Lane 3: AdhA G50D mutant (45 kDa). The target bands are highlighted with red triangles.

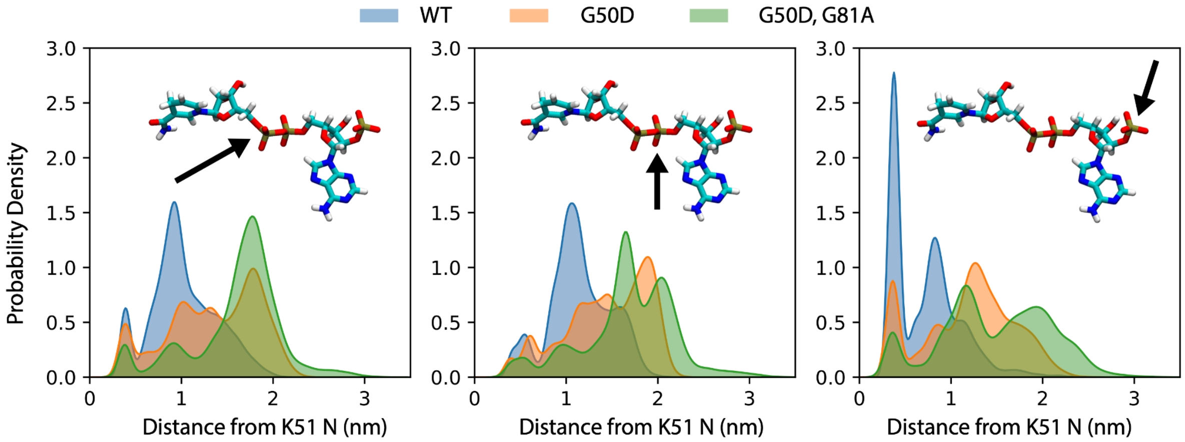

Supporting Figure S6. Histograms of distances between the nitrogen at the end of the sidechain of Lys-51 and each of the phosphorus atoms in NADPH for AdhA WT (blue), G50D (orange), and G50D + G81A (green). The phosphorus in question is indicated by an arrow inset within each plot. Histograms represent pooled data from eight independent 100-ns simulations of each variant.

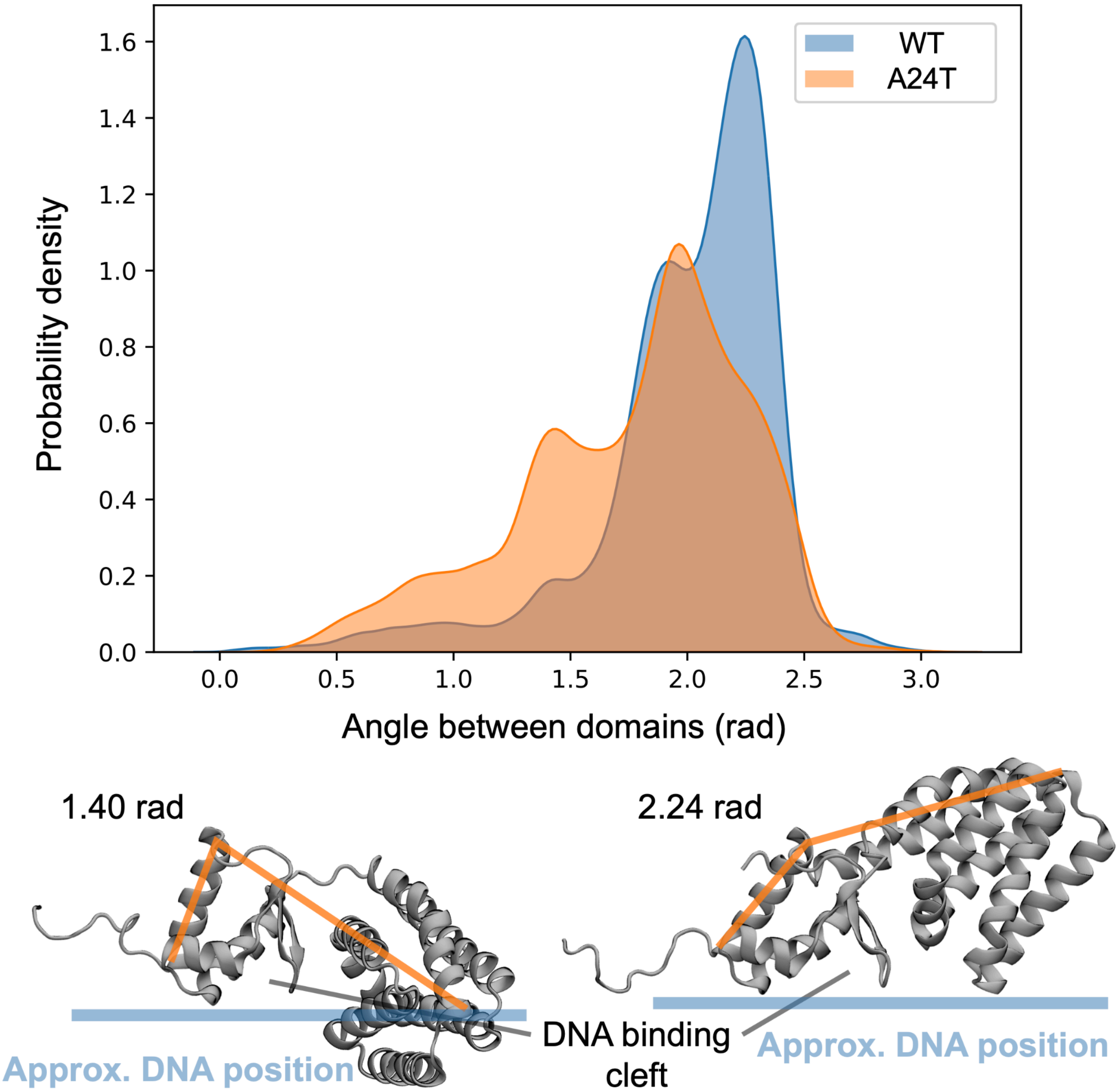

Supporting Figure S7. Histograms of angle between domains in GntR WT and A24T. The angle is computed between the alpha carbons of residues 13, 26, and 103, chosen because of their positions at the ends of relatively rigid alpha helices in each domain. Histograms represent pooled data from four independent 400-ns simulations of each variant. Snapshots at the bottom are both from WT simulations, overlaid with lines depicting the way the angle was computed and the approximate position that DNA would bind in.
